# Short-term association between ambient temperature and acute myocardial infarction hospitalizations for diabetes mellitus patients: A time series study

**DOI:** 10.1371/journal.pmed.1002612

**Published:** 2018-07-17

**Authors:** Holly Ching Yu Lam, Juliana Chung Ngor Chan, Andrea On Yan Luk, Emily Ying Yang Chan, William Bernard Goggins

**Affiliations:** 1 The Jockey Club School of Public Health and Primary Care, Faculty of Medicine, The Chinese University of Hong Kong, Prince of Wales Hospital, Hong Kong SAR; 2 Department of Medicine and Therapeutics, Faculty of Medicine, The Chinese University of Hong Kong, Prince of Wales Hospital, Hong Kong SAR; Africa Program, UNITED STATES

## Abstract

**Background:**

Acute myocardial infarction (AMI) is the leading cause of death among people with diabetes mellitus (DM) and has been found to occur more frequently with extreme temperatures. With the increasing prevalence of DM and the rising global mean temperature, the number of heat-related AMI cases among DM patients may increase. This study compares excess risk of AMI during periods of extreme temperatures between patients with DM and without DM.

**Methods:**

Distributed lag nonlinear models (DLNMs) were used to estimate the short-term association between daily mean temperature and AMI admissions (International Classification of Diseases 9th revision [ICD-9] code: 410.00–410.99), stratified by DM status (ICD-9: 250.00–250.99), to all public hospitals in Hong Kong from 2002 to 2011, adjusting for other meteorological variables and air pollutants. Analyses were also stratified by season, age group, gender, and admission type (first admissions and readmissions). The admissions data and meteorological data were obtained from the Hong Kong Hospital Authority (HA) and the Hong Kong Observatory (HKO).

**Findings:**

A total of 53,769 AMI admissions were included in the study. AMI admissions among DM patients were linearly and negatively associated with temperature in the cold season (cumulative relative risk [cumRR] [95% confidence interval] in lag 0–22 days (12 °C versus 24 °C) = 2.10 [1.62–2.72]), while those among patients without DM only started increasing when temperatures dropped below 22 °C with a weaker association (cumRR = 1.43 [1.21–1.69]). In the hot season, AMI hospitalizations among DM patients started increasing when the temperature dropped below or rose above 28.8 °C (cumRR in lag 0–4 days [30.4 versus 28.8 °C] = 1.14 [1.00–1.31]), while those among patients without DM showed no association with temperature. The differences in sensitivity to temperature between patients with DM and without DM were most apparent in the group <75 years old and among first-admission cases in the cold season. The main limitation of this study was the unavailability of data on individual exposure to ambient temperature.

**Conclusions:**

DM patients had a higher increased risk of AMI admissions than non-DM patients during extreme temperatures. AMI admissions risks among DM patients rise sharply in both high and low temperatures, with a stronger effect in low temperatures, while AMI risk among non-DM patients only increased mildly in low temperatures. Targeted health protection guidelines should be provided to warn DM patients and physicians about the dangers of extreme temperatures. Further studies to project the impacts of AMI risks on DM patients by climate change are warranted.

## Abstract 2

William Goggins and colleagues reveal an association between high and low ambient temperatures and acute myocardial infarction events for type 2 diabetes patients.

## Author summary

**Why was this study done?:**

- Mortality and morbidity from acute myocardial infarction (AMI) have been found to increase with both high and low temperatures.
- Few studies have been done on possible effect modification of this association by comorbidity with diabetes mellitus (DM), especially in warmer climates.

**What did the researchers do and find?:**

- A time series study was done using data on all 53,769 hospital admissions for AMI to public hospitals in Hong Kong from 2002 to 2011 as well as meteorological and pollutant data for the same time period.
- A modern regression method that allows for both nonlinear and lagged associations was used to assess the association between temperature and AMI admissions, stratified by DM status and controlling for other meteorological and pollutant variables and seasonal and long-term trends.
- Our results indicated that AMI admissions among those with DM history rose considerably more sharply at both high and low temperatures compared with admissions among subjects without a history of DM.

**What do these findings mean?:**

- Because AMI is often fatal and nonfatal occurrence can lead to complications such as heart failure, DM patients should be advised to avoid exposure to both high and low temperatures as much as possible.
- The combination of an increasing prevalence of DM and higher temperatures resulting from climate change may lead to more heat-related AMI occurrence in the future in the absence of mitigating actions.

## Introduction

Extreme ambient temperature has been reported to be associated with adverse health outcomes worldwide [1] and has also been linked to worsening of diabetes mellitus (DM) conditions and increased mortality [2–8]. A worldwide meta-regression study found a positive association between glucose intolerance and outdoor temperature [9]. Studies from the United States [2–4] and Sydney, Australia [5] found positive associations between temperature and DM-related complications and mortality, while studies from the Philippines [6] and China [7,8] found increased DM mortality at both high and low temperatures. Climate change, which is leading to higher average global temperatures [10], and the rising prevalence of DM have been suggested to pose a “dual threat” in increasing the disease burden [11].

Acute myocardial infarction (AMI) is one of the leading causes of death among people with DM [12], due to progressive narrowing of coronary arteries resulting in inadequate blood supply to the myocardium [13]. Previous studies have shown increasing AMI mortality and morbidity during both high and low temperatures. Studies from Cuba [14], Gothenburg, Sweden [15], Worcester, Massachusetts [16], Portugal [17], and Copenhagen, Denmark [18] found higher AMI risk at low temperatures, while studies from South Korea [19,20] and England and Wales [21,22] have reported increased AMI risk at both high and low temperatures. Patients with DM have also been found to be more vulnerable than non-DM patients to other health problems during extreme temperatures. Studies from England reported increasing general practitioner consultations among DM patients during periods of both high and low temperatures [11], while a study from Toronto reported a higher risk of cardiovascular emergency room visits among DM patients at high temperatures [23]. The temperature-sensitive natures of both DM and AMI and the higher vulnerability to other health problems among DM patients suggest the potential for a higher risk of temperature-related AMI admissions among DM patients. To the authors’ knowledge, only a few previous published studies have specifically compared AMI risk between DM and non-DM patients. A German study examined the association between myocardial infarction occurrence and ambient temperatures and found that higher AMI occurrence was associated with lower temperatures but that there was no effect modification of this association by history of DM [24]. A study from Worcester, Massachusetts found stronger increases in AMI occurrences among DM patients with decreases in apparent temperature during the cold season, although the difference between groups was not statistically significant (*p* = 0.11) [16]. The study found only a slightly stronger, modest increase in AMI occurrence with higher temperatures for the DM group during the hot season, and the difference between groups was not significant (*p* = 0.36) [16]. However, no such studies have been performed in subtropical or tropical climates.

In Hong Kong, previous studies have also reported increased cardiovascular [25,26] and AMI hospital admissions [27] at low temperatures as well as higher excess risks of natural mortality among DM patients during periods of both low and high temperatures [28]. The overall evidence points to a potential increased risk of AMI for DM patients during extremes of temperature. This study aimed to compare the relative risks of AMI admissions during extreme temperatures between DM and non-DM patients in Hong Kong, a city with a subtropical climate, using a retrospective time series approach.

## Methods

### Study design

Retrospective time series analyses were used to estimate the short-term association between temperature and AMI admissions in Hong Kong from 2002 to 2011. This study is part of a Health Medical Research Fund (HMRF)-funded project (the master project) in evaluating how DM modifies the short-term associations between ambient temperature and a series of cause-specific hospitalizations, including AMI, in Hong Kong. The protocol of the master study is shown in S1 Text.

### Environmental data

Records of daily mean temperature (°C), daily mean relative humidity (RH) percentage (%), daily mean solar radiation (MJ/m^2^), daily mean wind speed (km/h), and daily rainfall (mm) during the study period from the central monitoring station of the Hong Kong Observatory (HKO) were obtained from the website of the HKO. The HKO site was chosen because it is located in the densely populated city center close to where most of the Hong Kong population lives and because there were no missing data for this station during the study period. Records of daily mean concentration of respirable suspended particulates (RSPs; PM10 in μg/m^3^), sulphur dioxide (SO_2_ in μg/m^3^), nitrogen dioxide (NO_2_ in μg/m^3^), and ozone (O_3_ in μg/m^3^) from all general monitoring stations except Tap Mun—10 stations in total—were collected from the website of the Environmental Protection Department of Hong Kong for the same period and were averaged across the stations for each day. The records from Tap Mun, a general monitoring station in a remote area with a low population, and 3 roadside stations were excluded from the study because the records were less representative for the exposure of the general population [29]. If there was any missing record for a particular pollutant from a station on a day, the mean level of this pollutant on the day would be averaged across other stations with valid records. The meteorological data and air pollutants data can be accessed in the official website of the HKO (http://www.hko.gov.hk/contente.htm) and Environmental Protection Department (http://www.epd.gov.hk/epd/english/top.html).

### Admission data

Data on all hospital admissions for the years 1998 to 2011 from all public hospitals in Hong Kong were obtained from the Hong Kong Hospital Authority (HA). The HA data accounted for about 83% of overall hospitalizations in Hong Kong [26]. Principle diagnosis at discharge by International Classification of Diseases 9th revision (ICD-9) codes was used to identify AMI (ICD-9 = 410.00–410.99) cases. All AMI admissions between 2002 and 2011 were retrieved and examined for any previous admission records of DM, with records checked back to 1998, the earliest year for which complete data were available. Patients with admission records of DM (ICD-9 = 250.00–250.99) in any of the 10 first diagnoses at discharge prior to or concurrent with the AMI admission were considered to have DM. Each AMI admission was then classified into the DM or non-DM group.

### Statistical analysis

A combination of Poisson generalized additive models (GAMs) and distributed lag nonlinear models (DLNMs) [30] was used to examine associations between environmental variables and AMI hospitalizations while controlling for season, long-term trends, day of the week, and holidays. These models allow for both nonlinear and lagged associations, both of which are common in time series studies of environmental variables and health outcomes. The daily numbers of AMI admissions were regressed on all environmental exposures simultaneously in the initial model, with separate models fit for DM and non-DM groups. All analyses were performed separately for the cold season (November–April) and hot season (May–October) to minimize possible effect modification of exposure-outcome associations by season. Exposures considered included the daily means of temperature, RH, wind speed, the 4 air pollutants, and total daily solar radiation. Wind speed was square root–transformed, and PM10 was log-transformed to reduce skewness. Same-day rainfall, long-term time trend, seasonal trend, day of week, and holiday were adjusted in the models. Same-day rainfall was adjusted in models based on the hypothesis that heavy rain is likely to deter medical-help–seeking behavior [26]. Meteorological factors and air pollutants were modelled using crossbasis() functions created in the dlnm() package of R [31]. Maximum lags of 30 days for meteorological factors and 10 days for air pollutants were considered, and degrees of freedom (df) of 3, allowed for both exposure-outcome associations and lagged associations, were adopted for the models. Rainfall, long-term trend, and seasonality were adjusted in the models using splines in the mgcv() packages of R [32]. Based on the standard setting of 1 df for each year for long-term trend and 7 df for each year (i.e., df = 4 for each season with 6 months), the maximum df allowed for rainfall, long-term trend, and seasonal trend were 2, 10, and 4, respectively. More details on the statistical modeling are provided in S2 Text.

Temperature, RH, wind speed, and NO_2_ remained in the final model, while solar radiation, PM10, SO_3_, and O_3_ were dropped. The final model is shown below:

$$\begin{array}{l} \text{Log}\left(\text{E}\left[\text{daily}\;\text{no}.\;\text{of}\;\text{AMI}\;\text{admissions}\;\text{in}\;\text{DM}\left(\text{non}-\text{DM}\;\text{group}\right)\right]\right)=\text{cb}\left(\text{temp},\;\text{df}=3;\text{lag},\text{df}=3\right)+\text{cb}(\text{humid},\\ \text{df}=3;\text{lag},\text{df}=3)+\text{cb}\left(\text{sqrt}.\text{wind}\_\text{speed},\text{df}=3;\text{lag},\text{df}=3\right)+\text{cb}\left({\text{NO}}_{2},\text{df}=3;\text{lag},\text{df}=3\right)+\text{s}(\text{sqrt}.\text{Rain},\\ \text{maximum}\;\text{df}=2)+\text{s}\left(\text{long}\;\text{term}\;\text{trend}\;\text{maximum},\text{df}=10\right)+\text{s}\left(\text{seasonal}\;\text{trend},\text{maximum}\;\text{df}=4\right)+\\ \text{factor}\left(\text{DOW}\right)+\text{factor}\left(\text{Holiday}\right) \end{array}$$

- cb: crossbasis of independent variables built up with dlnm() package for DLNM in R
- s(): smoothing function of independent variables in mgcv() package for GAM in R
- factor(): indicator of categorical independent variables
- Long-term trend: day of study (1, 2, 3, …, 3,227)
- Seasonal trend: day of year (1, 2, 3, …, 365/366)
- DOW: day of week (1, 2, 3, …, 7)

The temperature at the 97th percentile during the hot season was taken to represent extreme high temperature, while that at the 3rd percentile during the cold season represented extreme low temperature. The reference points were chosen based on the nature of association observed. The temperature associated with the lowest risk in a U-shaped association, the minimum morbidity temperature (MMT), or—if a linear association was observed—the median temperature were used as the reference values for comparison. The association was considered statistically significant if the 95% confidence interval of relative risk did not include a relative risk = 1.0. To compare the relative risk of admissions between the DM and non-DM group, we examined the exposure-response curves for each group and calculated the relative risk ratio (RRR) and the corresponding 95% confidence interval using the approach suggested by Altman and Bland in 2003 [33], using the estimated relative risks and the corresponding 95% confidence intervals for the 2 groups (S2 Text).

Subgroup analyses were also performed, stratifying by age group (<75, ≥75), gender, and admission type (first AMI admissions, repeated admissions) to identify susceptible groups [21].

Maximum df = 4 and 14 for long-term trend, and df = 5 for crossbasis terms were applied as sensitivity analyses. Partial autocorrelation functions and residual plots were checked for appropriateness of models. The study was approved by the Survey and Behavioural Research Ethics committee of the Chinese University of Hong Kong.

## Results

### Descriptive summary

There were 53,796 AMI admissions during the study period (mean daily number of admissions = 14.73). Among these, 30.8% were among DM patients, 54.7% were admitted in the cold season, 61.7% were male, 48.9% were aged ≥75 years, and 75.2% were first-admission cases. The in-hospital mortality rates for all admissions, DM patients, and non-DM patients were 19.6%, 20.5%, and 19.2%, respectively (Table 1). The medians (interquartile range) of mean daily temperature and mean RH in the hot season were 27.80 °C (26.10 °C–29.10 °C) and 80.00% (75.00%–85.00%) (Table 2). The medians (interquartile range) of mean temperature and mean RH in the cold season were 19.30 °C (16.70 °C–21.80 °C) and 78% (69.00%–85.00%), respectively (Table 2)

**Table 1:** Descriptive statistics of public hospital admissions (ICD-9 = 410.00–410.99 for AMI; ICD-9 = 250.00–250.99 for DM) of Hong Kong SAR in 2002–2011.

AMI Admissions, *N* (%)							
	Cold			Hot			Total
	DM	Non-DM	*p*-Value	DM	Non-DM	*p*-Value	
**All**							
	9,356 (31.8%)	20,067 (68.2%)	-	7,211 (29.6%)	17,162 (70.4%)	-	53,796
**Gender**							
Female	4,516 (48.3%)	6,968 (34.7%)	<0.0005	3,404 (47.2%)	5,733 (33.4%)	<0.0005	20,621 (38.3%)
Male	4,840 (51.7%)	13,099 (65.3%)		3,807 (52.8%)	11,429 (66.6%)		33,175 (61.7%)
**Age group**							
<75 years	4,385 (46.9%)	10,092 (50.3%)	<0.0005	3,634 (50.4%)	9,385 (54.7%)	<0.0005	27,496 (51.1%)
≥75 years	4,971 (53.1%)	9,975 (49.7%)		3,577 (49.6%)	7,777 (45.3%)		26,300 (48.9%)
**Admission type**							
First admission	6,416 (68.6%)	15,685 (78.2%)	<0.0005	4,919 (68.2%)	13,415 (78.2%)	<0.0005	40,435 (75.2%)
Recurrent	2,940 (31.4%)	4,382 (21.8%)		2,292 (31.8%)	3,747 (21.8%)		13,361 (24.8%)
**Discharge status**							
Dead	1,945 (20.8%)	4,025 (20.1%)	0.16	1,451 (20.1%)	3,114 (18.1%)	<0.0005	10,535 (19.6%)
Discharged	7,411 (79.2%)	16,024 (79.9%)		5,760 (79.9%)	14,048 (81.9%)		43,261 (80.4%)

Abbreviations: AMI, acute myocardial infarction; DM, diabetes mellitus; ICD-9, International Classification of Diseases 9th revision.

**Table 2:** Descriptive statistics of daily mean temperature and daily mean RH in 2002–2011 in Hong Kong SAR.

Meteorological Variables	Percentile								
	1st	3rd	10th	25th	Median	75th	90th	97th	99th
**Cold Season (November–April)**									
Mean temperature (°C)	10.31	11.70	14.20	16.70	19.30	21.80	24.20	25.85	26.50
Maximum temperature (°C)	11.91	13.93	16.50	18.90	21.70	24.52	27.00	28.67	29.50
Minimum temperature (°C)	8.21	9.50	12.00	14.90	17.70	20.10	22.49	24.40	25.09
Mean RH (%)	41.00	48.00	60.00	69.00	78.00	85.00	90.00	94.00	95.00
Wind speed (km/hour)	5.31	7.03	11.50	17.18	23.6	29.8	35.30	41.43	46.46
Rainfall (mm)	0.00	0.00	0.00	0.00	0.00	0.01	2.10	12.17	34.06
NO_2_ (μg/ m^3^)	36.81	40.13	46.26	52.85	62.10	74.55	87.54	102.68	115.11
**Hot Season (May–October)**									
Mean temperature (°C)	22.44	23.60	24.80	26.10	27.80	29.10	29.80	30.40	30.80
Maximum temperature (°C)	24.24	25.30	26.80	28.40	30.20	31.90	32.80	33.70	34.30
Minimum temperature (°C)	20.64	21.90	23.09	24.50	25.80	27.20	28.00	28.58	28.90
Mean RH (%)	56.39	62.00	70.00	75.00	80.00	85.00	91.00	94.00	5.80
Wind speed (km/hour)	5.80	6.80	9.40	13.50	19.20	26.80	34.30	43.80	52.73
Rainfall (mm)	0.00	0.00	0.00	0.00	0.01	8.10	34.33	79.33	127.91
NO_2_ (μg/m^3^)	23.08	25.57	29.56	35.00	45.44	58.26	73.45	89.91	99.35

Abbreviation: RH, relative humidity.

Correlation analysis did not show high correlation between the 4 variables in both seasons (Table S2.1 in S2 Text).

### Regression results

#### Cold season

**Fig 1:**
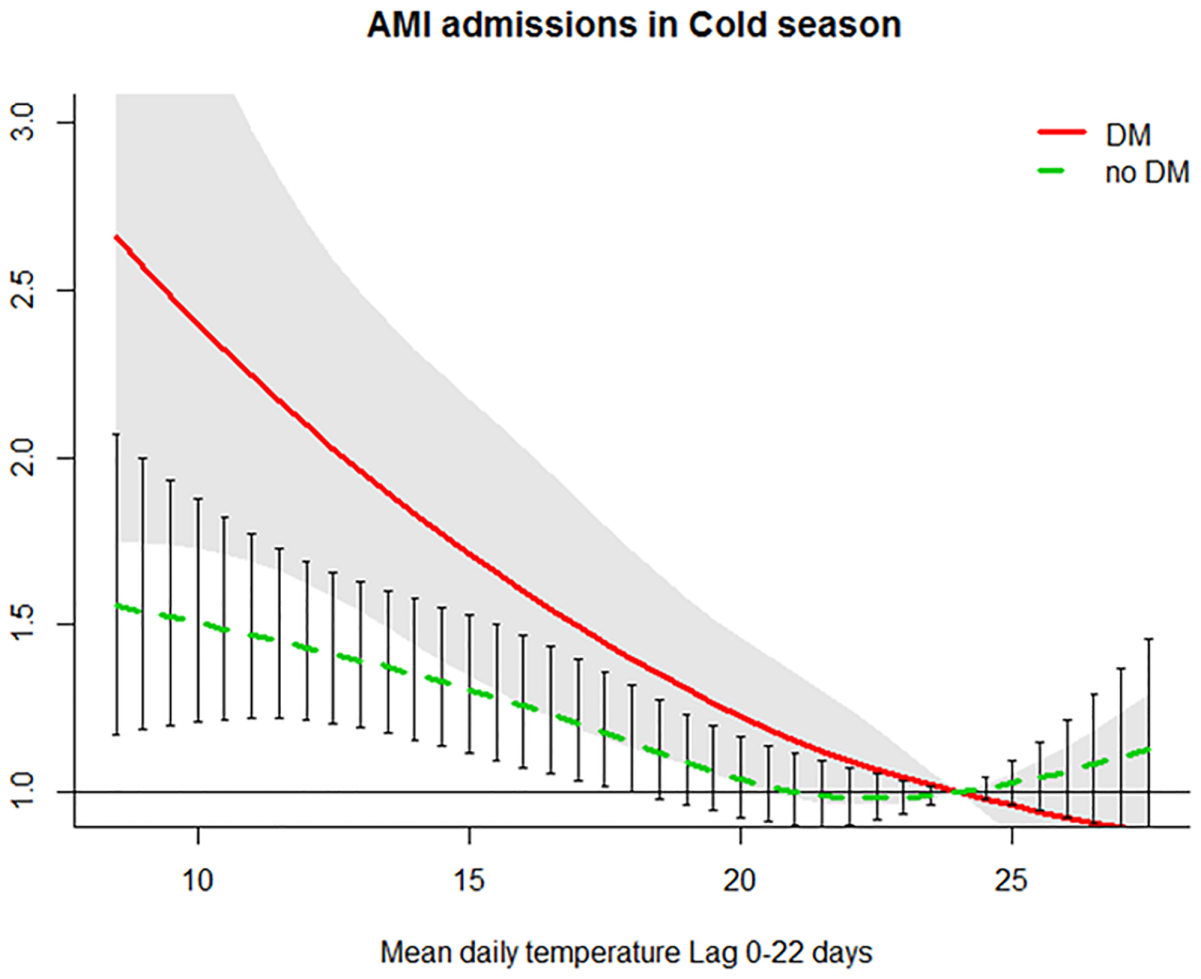
cumRR and the 95% confidence interval of AMI admissions among the DM group (red solid line and shaped area) and non-DM group (green dashed line and bars) in the cold season (November–April) in public hospitals of Hong Kong SAR in 2002–2011. AMI, acute myocardial infarction; cumRR, cumulative risk ratio; DM, diabetes mellitus.

**Fig 2:**
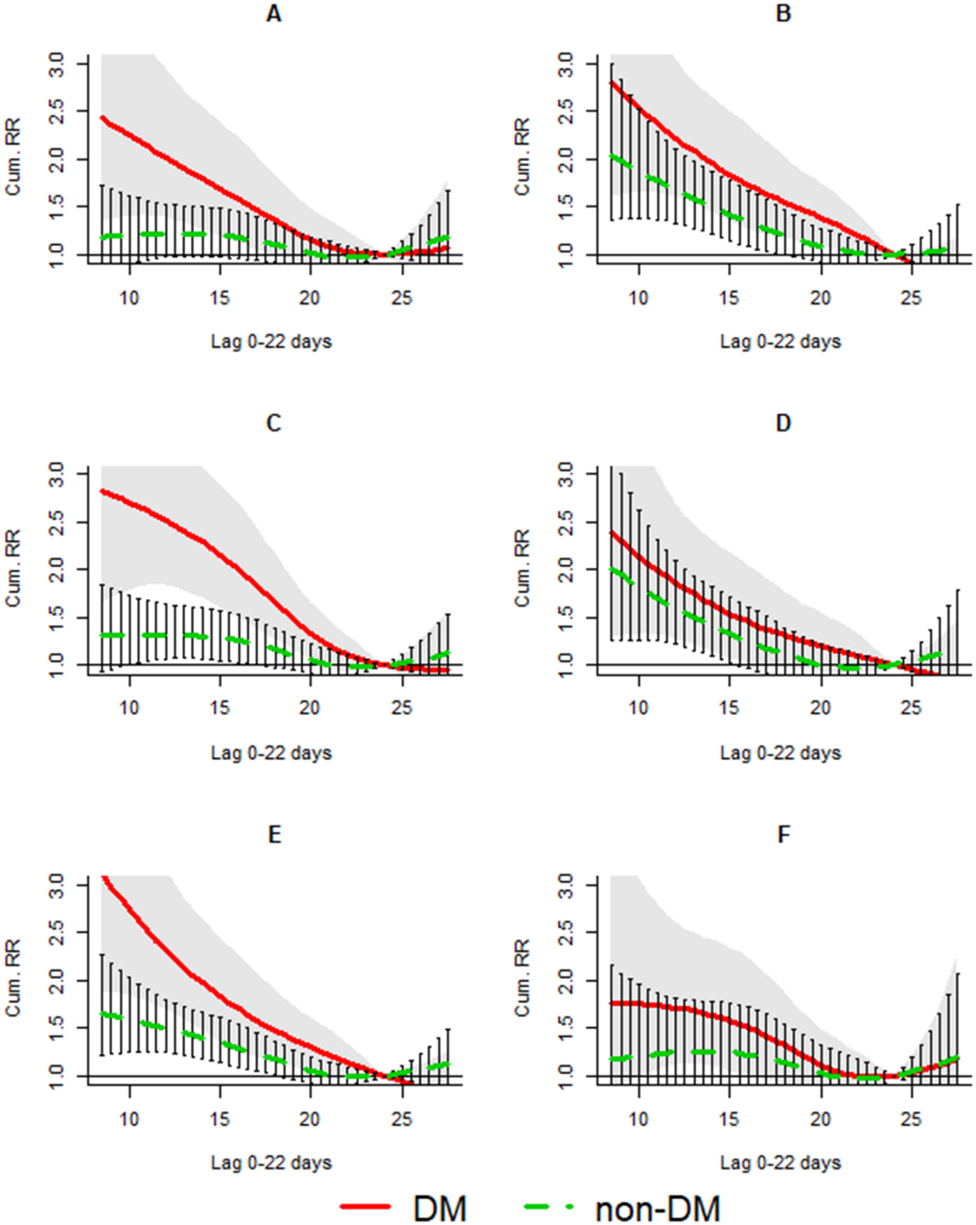
cumRR and the 95% confidence interval of AMI admissions among the DM group (red solid line and shaped area) and non-DM group (green dashed line and bars) in subgroups (A) <75 years, (B) ≥75, (C) male, (D) female, (E) first admissions, and (F) readmissions in the cold season (November–April) in public hospitals of Hong Kong SAR in 2002–2011. AMI, acute myocardial infarction; cumRR, cumulative risk ratio; DM, diabetes mellitus.

The number of AMI admissions generally increased when the temperature dropped. The association lasted for about 22 days for DM and non-DM patients. AMI admissions among DM patients increased sharply and linearly with decreasing temperatures throughout the range of temperatures observed in the cold season, while admissions among the non-DM group increased linearly only when the temperature dropped below 22 °C to 24 °C (Fig 1). In this season, the DLNM predicted the relative risk for every 0.5 °C. Therefore, although the 3rd percentile of temperature was 11.7 °C, we rounded it up to 12 °C to represent the extreme low temperature in this season. The respective cumulative RRs (cumRRs) (95% confidence interval) in lag 0–22 days (12 °C versus 24 °C) for the DM and non-DM groups were 2.10 (1.62–2.72) and 1.43 (1.21–1.69) (Table 3). The association with low temperature was significantly stronger among the DM group, with an RRR (95% confidence interval) of 1.46 (1.07–1.99) (Table 3). Subgroup analyses showed that among the non-DM group, older age (versus <75), female (versus male), and first-admission cases (versus recurrent) demonstrated stronger increased risk with cool temperatures. In general, DM patients had stronger increased risk with cooler temperatures than non-DM patients in every subgroup (Table 3). However, among the DM group, the increased risk during low temperatures was stronger for the male group. The differences in sensitivity to low temperatures between the DM and non-DM groups were more apparent among the <75 group (RRR = 1.66 [1.07–2.59]), males (RRR = 1.91 [1.30–2.80]), and first-admission cases (RRR = 1.55 [1.08–2.22]) (Table 3) (Fig 2). The different sensitivities to temperature between gender and admissions type remained after analyses were further stratified by age group.

**Table 3:** cumRR of AMI admissions during extreme temperatures and RRR between the DM and non-DM group in the cold season (November–April) in public hospitals of Hong Kong SAR in 2002–2011.

	DM	Non-DM	RRR (95% confidence interval) (DM versus non-DM)	*p*-Value of RRR
	cumRR (95% confidence interval)	cumRR (95% confidence interval)		
Cold season (November–April) lag 0–22 days; 12 °C versus 24 °C				
All AMI	2.10 (1.62–2.72)	1.43 (1.21–1.69)	1.46 (1.07–1.99)	0.02
<75	2.02 (1.38–2.94)	1.21 (0.96–1.53)	1.66 (1.07–2.59)	0.02
≥75	2.22 (1.61–3.05)	1.67 (1.32–2.12)	1.33 (0.89–1.97)	0.16
Female	1.86 (1.28–2.69)	1.59 (1.20–2.11)	1.17 (0.73–1.86)	0.52
Male	2.52 (1.83–3.45)	1.32 (1.06–1.64)	1.91 (1.30–2.80)	<0.005
First admission	2.32 (1.70–3.16)	1.50 (1.25–1.80)	1.55 (1.08–2.22)	0.02
Recurrent	1.71 (1.12–2.61)	1.24 (0.85–1.81)	1.38 (0.78–2.43)	0.27

Abbreviations: AMI, acute myocardial infarction; cumRR, cumulative relative risk; DM, diabetes mellitus; RRR, relative risk ratio.

#### Hot season

**Fig 3:**
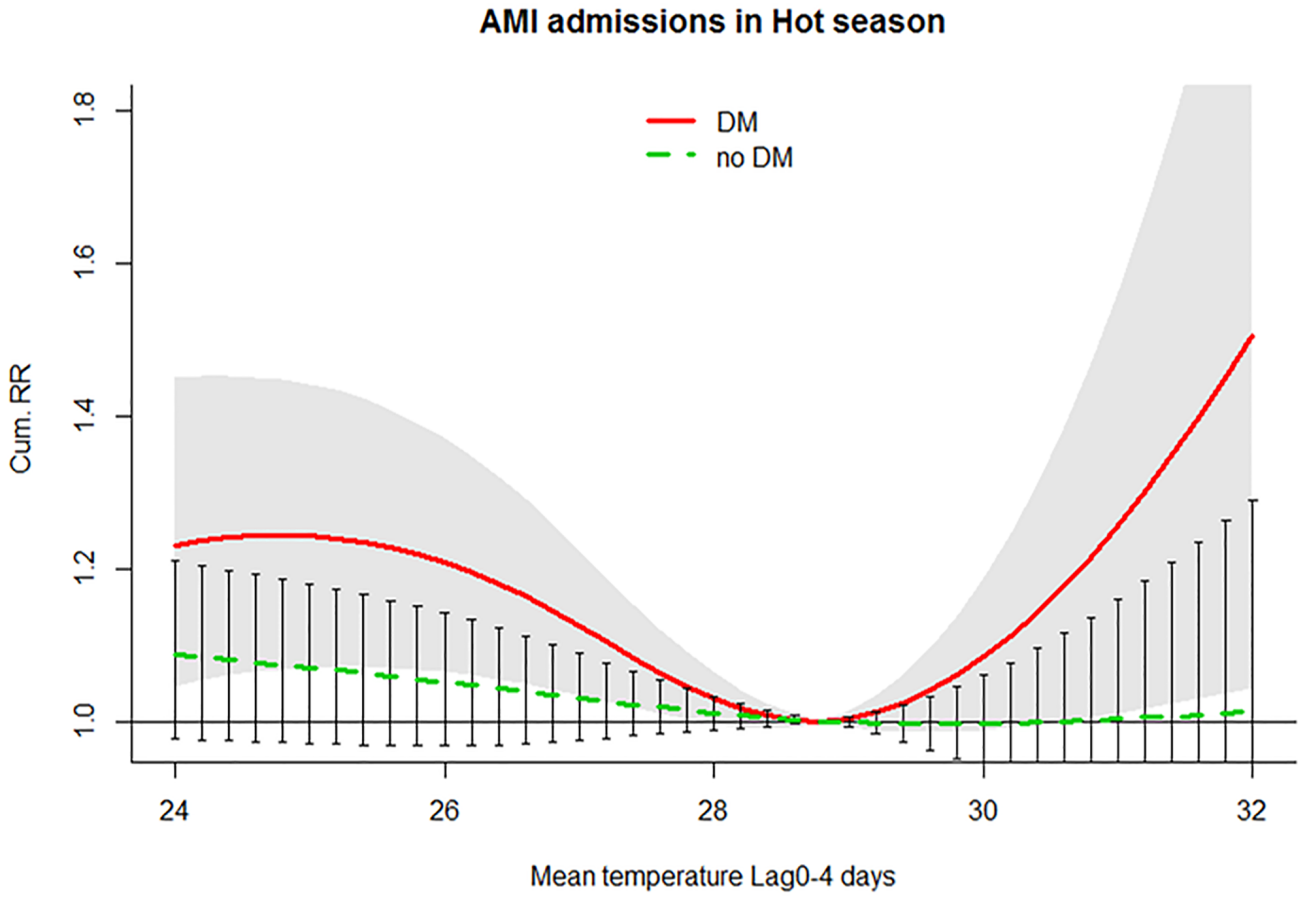
cumRR and the 95% confidence interval of AMI admissions among the DM group (red solid line and shaped area) and non-DM group (green dashed line and bars) in the hot season (May–October) in public hospitals of Hong Kong SAR in 2002–2011. AMI, acute myocardial infarction; cumRR, cumulative risk ratio; DM, diabetes mellitus.

**Fig 4:**
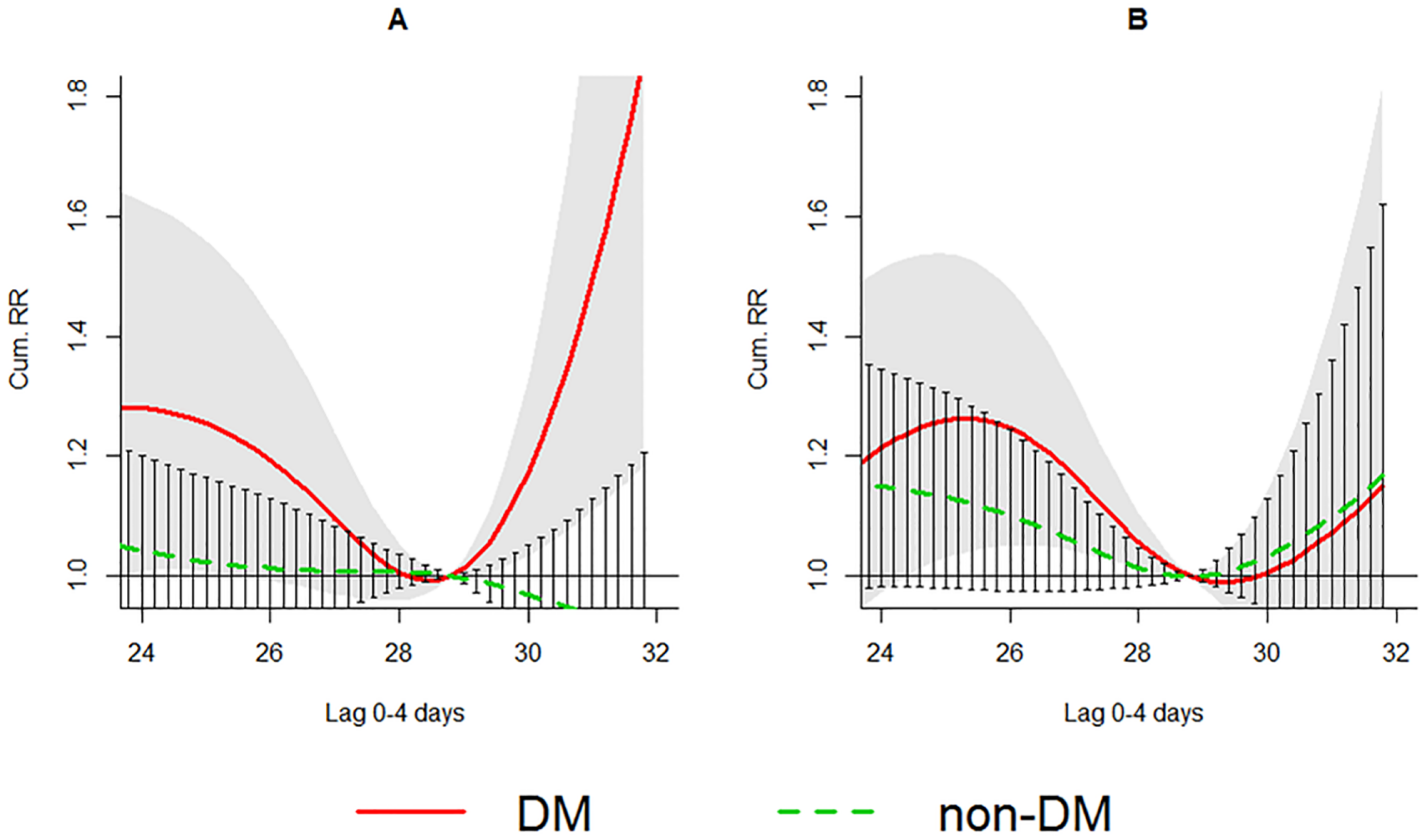
cumRR and the 95% confidence interval of AMI admissions among the DM group (red solid line and shaped area) and non-DM group (green dashed line and bars) in subgroups (A) <75 years and (B) ≥75 years in the hot season (May–October) in public hospitals of Hong Kong SAR in 2002–2011. AMI, acute myocardial infarction; cumRR, cumulative risk ratio; DM, diabetes mellitus.

A U-shaped association between temperature and AMI admissions was detected among DM patients, with minimum morbidity at about 28.8 °C (Fig 3). The excess risk associated with high temperatures peaked on the same day and persisted for about 4 days. The cumRR in lag 0–4 days (30.4 °C versus 28.8 °C) was 1.14 (1.00–1.31) (Table 4). For non-DM patients, the number of AMI admissions did not rise with high temperatures (cumRR = 1.00 [0.91–1.10]) (Table 4). The RRR comparing cumRR between the DM and non-DM group at 30.4 °C (versus 28.8 °C) was 1.14 (0.97–1.34). Among non-DM patients, there were no obvious differences in relative risks between age groups, genders, and admission types (Table 4). Similar to the results in the cold season, the relative risk of admissions of DM patients were generally higher than those of non-DM patients except for the ≥75 age group (Table 4). The increased risks of the DM group were strongest for subjects <75 years old and were similar between genders and admission types. A significant difference in sensitivity to high temperatures between the DM and non-DM group was observed in subjects <75 years (RRR = 1.33 [1.07–1.67]) (Table 4) (Fig 4).

**Table 4:** cumRR of AMI admissions during extreme temperatures and RRR between the DM and non-DM group in the hot season (May–October) in public hospitals of Hong Kong SAR in 2002–2011.

	DM	Non-DM	RRR (95% confidence interval) (DM versus non-DM)	*p*-value of RRR
	cumRR (95% confidence interval)	cumRR (95% confidence interval)		
Hot season (May–October) lag 0–4 days				
**30.4 °C versus 28.8 °C**				
All AMI	1.14 (1.00–1.31)	1.00 (0.91–1.10)	1.14 (0.97–1.34)	0.12
<75	1.28 (1.06–1.55)	0.96 (0.85–1.08)	1.33 (1.07–1.67)	0.01
≥75	1.03 (0.85–1.24)	1.06 (0.92–1.21)	0.97 (0.77–1.23)	0.81
Female	1.16 (0.95–1.42)	1.01 (0.86–1.18)	1.15 (0.89–1.48)	0.29
Male	1.13 (0.93–1.36)	0.99 (0.89–1.11)	1.14 (0.92–1.42)	0.24
First admission	1.13 (0.96–1.34)	0.98 (0.89–1.08)	1.15 (0.95–1.40)	0.15
Recurrent	1.15 (0.92–1.46)	1.08 (0.89–1.32)	1.07 (0.79–1.45)	0.66
**23.6 °C versus 28.8 °C**				
All AMI	1.22 (1.02–1.44)	1.09 (0.97–1.22)	1.12 (0.91–1.38)	0.29
<75	1.28 (1.00–1.65)	1.05 (0.91–1.22)	1.22 (0.91–1.63)	0.18
≥75	1.18 (0.94–1.49)	1.15 (0.98–1.36)	1.03 (0.77–1.36)	0.86
Female	1.40 (1.09–1.80)	1.19 (0.98–1.45)	1.18 (0.86–1.62)	0.32
Male	1.07 (0.85–1.36)	1.05 (0.92–1.20)	1.02 (0.78–1.33)	0.89
First admission	1.20 (0.97–1.47)	1.23 (1.00–1.27)	0.98 (0.77–1.24)	0.84
Recurrent	1.23 (0.92–1.63)	1.00 (0.79–1.27)	1.23 (0.85–1.78)	0.27

Abbreviations: AMI, acute myocardial infarction; cumRR, cumulative relative risk; DM, diabetes mellitus; RRR, relative risk ratio.

AMI risks in both the DM and non-DM groups also increased when the temperature dropped below 28.8 °C. The association was stronger for the DM group (cumRR [23.6 °C versus 28.8 °C; lag 0–4days] = 1.22 [1.02–1.44]) than the non-DM group (cumRR = 1.09 [0.97–1.22]), but the difference was not significant (Table 4). Sensitivity analysis using df = 4 or 14, and using df = 5 for exposure and lag parameters gave results that were consistent with those from the main analysis. The results of the sensitivity analyses can be found in S3 Text.

## Discussion

While AMI admissions in Hong Kong increased when temperatures dropped in the cold season for both DM and non-DM patients, admissions among DM patients showed a higher temperature threshold and a significantly stronger association with temperature in the cold season. In the hot season, the number of AMI admissions among DM patients increased significantly with rising temperatures above 28.8 °C, but no increased risk with high temperatures was seen for the non-DM group. The increased relative risk for DM patients with lower temperatures was higher than for the non-DM group for all subgroups. The greater sensitivity to low temperature for DM patients was more apparent in the group below 75 years old, males, and first-admission cases. In the hot season, no obvious increased relative risk was observed among non-DM patients in all subgroups at high temperatures. DM patients demonstrated higher relative risks than non-DM patients in most of the subgroups. Patients <75 showed statistically significantly different sensitivities to high temperatures between the DM and non-DM groups in the hot season, with a higher increased relative risk for the DM group.

A previous temperature–AMI-admissions study in Hong Kong found that more admissions were associated with lower temperatures but did not find an increase at higher temperatures [27]. The results are consistent with the findings for the non-DM patients in this study. Our finding that AMI hospitalizations among DM patients showed greater sensitivity to ambient temperature, particularly during the cold season, is consistent with the results from a previous study in Worcester, Massachusetts [16], a region with a humid continental climate. Compared with Hong Kong, Worcester has a cooler summer and much colder winter. As reported in the Worcester study, the daily temperature at the 5th/95th percentile was −10.0 °C/10.3 °C during the cold months and was 4.4 °C/24.2% in the warm months [16]. This indicated that DM could modify the temperature–AMI association in both subtropical and continental regions. However, our results were different from that of a previous study from Augsburg, Germany that found a modest association of higher AMI occurrence at lower temperatures but no substantial or significant difference in the association between DM and non-DM patients [24]. Augsburg has a relatively tempered climate and a larger temperature variation than Worcester and Hong Kong. Although the daytime maximum can be quite high, as evidenced by a maximum daily temperature of 39.2 °C reported for their study, the maximum mean daily temperature during their 10-year study period was only 27.9 °C [24], below the threshold at which our study found high temperature associations. While a lower threshold for heat effects would be expected in a cooler climate, the maximum daily minimum temperature reported by their study was 19.8 °C [24], indicating that nighttime relief from high temperatures was consistently present. This is not the case in Hong Kong, where summertime minimum temperatures are frequently >27 °C. In addition, while the Augsburg study adopted the year-round analysis approach, the Worcester study and our study performed seasonal analysis. Seasonal analysis may help reduce the effect modification by seasonal factors that may bias the association. For instance, in regions that have central heat in the cold months only, the same temperature in different seasons may be associated with different health outcomes because people will be protected by the heating system during the cold months. Differences in genetics and/or behavior between the two populations may have also contributed to the difference in findings.

To our knowledge, only a few studies have reported greater sensitivity to extreme temperatures among DM patients for other health outcomes. A large cohort study of Hong Kong elderly [28] found that natural mortality increased more at both high and low temperatures among patients with DM than among those with no comorbidities or those with other comorbidities, including chronic obstructive pulmonary disease (COPD) and circulatory diseases. A study of emergency room admissions from Toronto [34] found greater increases in admission rates for cardiovascular disease in DM patients with extreme high temperatures than in those without DM, but the risk among DM patients was not increased in low temperatures. A study from England reported that general practitioner consultations rose among DM patients at both high and low temperatures [11]. The authors concluded that the increase in consultations during extreme heat was likely to be specific to DM patients because previous work indicated no increase in overall consultations during the 2013 heat wave. That study also reported more heat-related consultations for cardiovascular disease among DM patients, but they did not perform subgroup analyses for cold effect [11].

The results of our study agreed with those from previous studies that DM patients were more vulnerable during high temperatures than non-DM patients, especially for cardiovascular risks. Patients with DM have endothelial dysfunction and poor skin blood flow, which can compromise thermoregulation and alter hemostasis, which can increase cardiac stress and risk of cardiovascular disease during extreme temperatures [35].

Our study found that the increased risk of temperature-associated AMI for DM patients compared with non-DM patients was consistently stronger among patients <75 years old—compared with those ≥75—and in fact, during the hot season, there was almost no difference observed between the DM and non-DM groups among those ≥75. One possible explanation for this is that DM may remove the protective effect of younger age, especially during periods of extreme temperatures, by compromising thermoregulation ability at a relatively younger age, although this hypothesis requires further examination. A previous study has also found that the effects of DM on cardiovascular risks were less apparent among older adults and that cardiovascular risks were not different between older adults with and without DM [36]. Although the older groups (with and without DM) were more likely to have AMI at extreme temperatures, there was a much larger difference in sensitivity to temperature between DM and non-DM groups in the younger group. Thus, while attention should be paid to older patients regardless of DM status during extreme temperatures, such caution should be applied to all patients with DM irrespective of age. Our study also found that the first-admission events were more sensitive to temperature, which may be due to increased awareness and medical attention following previous admission for AMI.

The results of the non-DM analysis in this study agreed with a previous study in Hong Kong that found that risk of overall AMI admissions increased when temperatures dropped below 24 °C [27]. Consistent associations with low temperatures have also been reported in other regions, including Cuba [14], Gothenburg, Sweden [15], Worcester, Massachusetts [16], Portugal [17], and Augsburg, Germany [24] as well as Copenhagen and Denmark [18]. Studies from Korea [19,20] and England and Wales [21,22] have, however, reported that AMI risks increased at both high and low temperatures. Besides the effects of diabetes, genetics, and behavior, the heterogeneous temperature–AMI associations reported might be related to the differences in climate and methodology used for study.

Another possible reason that some studies did not find an association between AMI risk and high temperatures is that, for some populations and climates, high-temperature effects on AMI may be very short-term. A study from England and Wales [22] found that, while the effect of low temperatures on AMI risk could be captured in daily study, the effect of high temperatures could only be captured by an hourly-based study due to the much shorter lagged effect. This might be another reason why some previous studies assessing daily-temperature effects on AMI risks—including the previous Hong Kong AMI study [27] and the non-DM group analyses in this study—only found pronounced cold effects. Future studies evaluating hourly-based lagged effects of high temperatures should be conducted in warmer regions to test the hypothesis. Genetics and behavior might also contribute to the different associations because studies from Korea have found associations between high temperatures and AMI risk [19,20].

The possible mechanisms of increasing AMI risk during low temperatures include higher blood pressure, cardiac hypertrophy, increased platelet counts, and blood viscosity [37–39]. Low temperature has been found to be associated with increased low-density lipoprotein cholesterol and decreased high-density lipoprotein cholesterol, which might contribute to increased AMI risk during low temperatures [40]. Heat exposures may cause physiological changes such as increasing heart rate, blood viscosity, and platelet and red cell counts [41]. At high temperatures, vessel dilation and blood flow from vital organs to skin surface for cooling may also increase cardiac strain and the risk of AMI [42].

According to the Fifth Assessment Report of the Intergovernmental Panel on Climate Change, the mean global temperature is increasing, while the number of cold days are very likely to decrease [10]. The dual phenomena of global warming and the global epidemic of diabetes may lead to increasing numbers of heat-related AMI events among DM patients. While climate change may reduce the frequency of low-temperature–related AMI events, the increasing prevalence of DM in the population and the possibility that DM patients are more sensitive to cold temperatures makes this uncertain.

It is important to note that, while we present our findings in terms of absolute temperatures, in general, ambient temperatures relative to the norm for a particular climate have been found to be very important in terms of impact on health outcomes. Thus, while a U-shaped association between health outcomes and daily ambient temperatures are usually found in time series studies, the MMTs—as well as the thresholds above and below which high- and low-temperature effects are noted—vary considerably between areas with different climates [1], with cooler climates generally having lower MMTs and thresholds for both high- and low-temperature effects due to local adaption to climate. Therefore, the specific absolute mean daily temperatures identified as MMTs and thresholds in this study should not be taken as universally applying to areas with different climates. In addition, diurnal (same-day) temperature range (DTR) may also be associated with health outcomes [43–47]. In this study, we performed a sensitivity analysis including diurnal temperature, daily maximum temperature–daily minimum temperature, in our models. We found that this variable was not associated with AMI admissions for either group in the hot season, and only very weakly and negatively associated with AMI for the DM group only in the cold season. In addition, the inclusion of this variable did not affect our main results. The lack of association may be due to the generally small DTR observed in Hong Kong, which is also fairly consistent between days, due to its high humidity and proximity to the ocean. In other areas with larger diurnal temperature variation and/or greater inter-day variation in DTR, the effects of this variable may be important.

Our study has several limitations. The types of MI classified by elevation of ST segment on electrocardiogram, medication records and presence of other co-morbidities were not considered due to unavailable data and the focus of the study. These factors may influence the nature of the temperature–AMI association among DM patients. Thus, this study was not able to reflect the effect of disease severity on temperature–AMI association. Secondly, this study used principle diagnosis at discharge to identify AMI cases and assumed no diagnosis errors. Cases that admitted to hospitals due to other causes but were diagnosed with AMI during hospitalizations were included, while those admitted due to AMI but were resulting in other complications were not considered in this study. The prior would cause overestimation of temperature–AMI association, while the latter would bias the association in a negative direction. Moreover, the exposure level to environmental factors was assumed to be the same for the whole population. The exposure level, however, might vary according to geographical area of residence. While meteorological data collected from a single station in the city center will not completely reflect the ambient temperature exposure for all Hong Kong residents, Hong Kong is quite small geographically and so large differences in mean daily temperatures are generally not observed. As the between-group difference in associations with high temperatures was not statistically significant, it is possible that the stronger association among the DM patients could be due to chance. Although we also note that the stronger heat association for DM patients <75 years old was significant. Finally, several independent variables were considered in the same model which might raise the problem of type I error rate inflation due to multiple testing. The results presented in this paper were not adjusted for multiple testing therefore attention should be paid when interpreting the results.

## Conclusion

Our study found that AMI admissions increased more sharply for DM patients relative to non-DM patients during extreme temperatures, with between-group differences being particularly strong for low-temperature associations. The results showed robust association of increased risk of AMI hospitalizations among DM patients at low temperatures in both seasons and high temperatures in the hot season. By contrast, we only found mildly increased risk among non-DM patients at low temperatures, and no increase at high temperatures. The difference in sensitivity of admission numbers to temperature between DM and non-DM patients were more obvious for patients younger than 75 years old. Findings of this study should be taken into account when drafting targeted health policy against extreme temperatures and planning patient care for people with diabetes. Further studies from regions with differing climates examining effect modification of AMI–temperature associations by DM status are needed. Future studies projecting the possible effects of rising temperatures on AMI incidence among DM patients should be considered.

## Supporting information

S1 Text Proposal of the master project (HMRF DM project 2013). (PDF)

S2 Text Model selection and correlation table. (DOCX)

S3 Text Results of sensitivity analysis. (DOCX)

S1 RECORD Checklist The RECORD statement. (DOCX)

## Abbreviations

AMI: acute myocardial infarction
COPD: chronic obstructive pulmonary disease
cumRR: cumulative relative risk
df: degrees of freedom
DLNM: distributed lag nonlinear model
DM: diabetes mellitus
DTR: diurnal temperature range
GAM: generalized additive model
HA: [Hong Kong] Hospital Authority
HKO: Hong Kong Observatory
HMRF: Health Medical Research Fund
ICD-9: International Classification of Diseases 9th revision
MMT: minimum morbidity temperature
RH: relative humidity
RRR: relative risk ratio
RSP: respirable suspended particulate

## References

[1] Gasparrini A, Guo Y, Hashizume M, Lavigne E, Zanobetti A, Schwartz J, et al Mortality risk attributable to high and low ambient temperature: A multicountry observational study. Lancet. 2015;386(9991):369–75. 10.1016/S0140-6736(14)62114-0 26003380 PMC4521077

[2] Basu R, Pearson D, Malig B, Broadwin R, Green R. The effect of high ambient temperature on emergency room visits. Epidemiology. 2012 11;23(6):813–20. 10.1097/EDE.0b013e31826b7f97 23007039

[3] Green RS, Basu R, Malig B, Broadwin R, Kim JJ, Ostro B. The effect of temperature on hospital admissions in nine California counties. Int J Public Health. 2010 4;55(2):113–21 10.1007/s00038-009-0076-0 19771392

[4] Schwartz J. Who is sensitive to extremes of temperature?: A case-only analysis. Epidemiology. 2005 16(1):67–72. 15613947 10.1097/01.ede.0000147114.25957.71

[5] Vaneckova P, Bambrick H. Cause-specific hospital admissions on hot days in Sydney, Australia. PLoS ONE. 2013 1;8(2):e55459 10.1371/journal.pone.0055459 23408986 PMC3567089

[6] Seposo XT, Dang TN, Honda Y. How Does Ambient Air Temperature Affect Diabetes Mortality in Tropical Cities? Int J Environ Res Public Health. 2017;14(385). 10.3390/ijerph14040385 PMC5409586 28379204

[7] Yang J, Yin P, Zhou M, Ou C-Q, Li M, Liu Y, et al The effect of ambient temperature on diabetes mortality in China: A multi-city time series study. Sci Total Environ. 2016 2 1;543(Pt A):75–82. 10.1016/j.scitotenv.2015.11.014 26580729

[8] Li Y, Lan L, Wang Y, Yang C, Tang W, Cui G, et al Extremely cold and hot temperatures increase the risk of diabetes mortality in metropolitan areas of two Chinese cities. Environ Res. 2014;134:91–7. 10.1016/j.envres.2014.06.022 25086705

[9] Blauw LL, Aziz NA, Tannemaat MR, Blauw CA, de Craen AJ, Pijl H, et al Diabetes incidence and glucose intolerance prevalence increase with higher outdoor temperature. BMJ Open Diabetes Res Care. 2017;5:e000317 10.1136/bmjdrc-2016-000317 28405341 PMC5372132

[10] IPCC2014. Climate Change 2014 Synthesis Report Summary Chapter for Policymakers. 2014.

[11] Hajat S, Haines A, Sarran C, Sharma A, Bates C, Fleming LE. The effect of ambient temperature on type- 2-diabetes: case-crossover analysis of 4 + million GP consultations across England. Envionmental Health. 2017;16(73):1–8. 10.1186/s12940-017-0284-7 PMC5506566 28701216

[12] Leon BM. Diabetes and cardiovascular disease: Epidemiology, biological mechanisms, treatment recommendations and future research. World J Diabetes. 2015;6(13):1246 10.4239/wjd.v6.i13.1246 26468341 PMC4600176

[13] Mendis S, Thygesen K, Kuulasmaa K, Giampaoli S, Ma M. World Health Organization definition of myocardial infarction: 2008–09 revision. 2011; (October 2010):139–46. 10.1093/ije/dyq165 20926369

[14] Rivero A, Bolufé J, Ortiz PL, Rodríguez Y, Reyes MC. Influence of Climate Variability on Acute Myocardial Infarction Mortality in Havana, 2001–2012. MEDICC Rev. 2015;17(2):14–9. 26027582 10.37757/MR2015.V17.N2.5

[15] Barregard L, Sallsten G. Association between Ambient Temperature and Acute Myocardial Infarction Hospitalisations in Gothenburg, Sweden: 1985–2010. PLoS ONE. 2013;8(4). 10.1371/journal.pone.0062059 PMC3639986 23646115

[16] Madrigano J, Mittleman MA, Baccarelli A, Goldberg R, Melly S, von Klot S, et al Temperature, myocardial infarction, and mortality: effect modification by individual- and area-level characteristics. Epidemiology. 2014;24(3):439–46. 10.1097/EDE.0b013e3182878397 PMC4037287 23462524

[17] Vasconcelos J, Freire E, Almendra R, Silva GL. The impact of winter cold weather on acute myocardial infarctions in Portugal. Environ Pollut. 2013;183:14–8. 10.1016/j.envpol.2013.01.037 23410618

[18] Wichmann J, Ketzel M, Ellermann T, Loft S. Apparent temperature and acute myocardial infarction hospital admissions in Copenhagen, Denmark: a case-crossover study. Environ Health. 2012; 11:19 10.1186/1476-069X-11-19 22463704 PMC3353865

[19] Lee S, Lee E, Park MS, Kwon BY, Kim H, Jung DH, et al Short-Term Effect of Temperature on Daily Emergency Visits for Acute Myocardial Infarction with Threshold Temperatures. PLoS ONE. 2014;9(4). 10.1371/journal.pone.0094070 PMC4000206 24770787

[20] Kwon BY, Lee E, Lee S, Heo S, Jo K, Kim J, et al Vulnerabilities to Temperature Effects on Acute Myocardial Infarction Hospital Admissions in South Korea. Int J Environ Res Public Health. 2015;37:14571–88. 10.3390/ijerph121114571 PMC4661668 26580643

[21] Bhaskaran K, Hajat S, Haines A, Herrett E, Wilkinson P, Smeeth L. Short term effects of temperature on risk of myocardial infarction in England and Wales: time series regression analysis of the Myocardial Ischaemia National Audit Project. BMJ. 2010;341:c3823 10.1136/bmj.c3823 20699305 PMC2919679

[22] Bhaskaran K, Armstrong B, Hajat S, Haines A, Wilkinson P, Smeeth L. Heat and risk of myocardial infarction: hourly level case-crossover analysis of MINAP database. BMJ. 2012;345:e8050 10.1136/bmj.e8050 23243290 PMC3521646

[23] Lavigne E, Gasparrini A, Wang X, Chen H, Yagouti A, Fleury MD, et al Extreme ambient temperatures and cardiorespiratory emergency room visits: assessing risk by comorbid health conditions in a time series study. Environ Health. 2014 1;13(1):5 10.1186/1476-069X-13-5 24484632 PMC3922624

[24] Wolf K, Schneider A, Breitner S, von Klot S, Meisinger C, Cyrys J, et al Epidemiology and Prevention Air Temperature and the Occurrence of Myocardial Infarction in Augsburg, Germany. Circulation. 2009;120:735–42. 10.1161/CIRCULATIONAHA.108.815860 19687361

[25] Tian L, Qiu H, Sun S, Lin H. Emergency Cardiovascular Hospitalization Risk Attributable to Cold Temperatures in Hong Kong. Circ Cardiovasc Qual Outcomes. 2016 3;9(2):135–42. 10.1161/CIRCOUTCOMES.115.002410 26933049

[26] Chan EYY, Goggins WB, Yue SK, Lee P. Hospital admissions as a function of temperature, other weather phenomena and pollution levels in an urban setting in China. Bull World Heal Organ. 2013; (April):576–84. 10.2471/BLT.12.113035 PMC3738307 23940405

[27] Goggins WB, Chan EYY, Yang C. Weather, pollution, and acute myocardial infarction in Hong Kong and Taiwan. Int J Cardiol. 2013;168(1):243–9. 10.1016/j.ijcard.2012.09.087 23041014

[28] Sun S, Tian L, Qiu H, Chan K-P, Tsang H, Tang R, et al The influence of pre-existing health conditions on short-term mortality risks of temperature: Evidence from a prospective Chinese elderly cohort in Hong Kong. Environ Res. 2016 7;148:7–14. 10.1016/j.envres.2016.03.012 26994463

[29] Lam HC-Y, Li AM, Chan EY-Y, Goggins WB. The short-term association between asthma hospitalisations, ambient temperature, other meteorological factors and air pollutants in Hong Kong: a time-series study. Thorax. 2016 6 24;1–13. 10.1136/thoraxjnl-2015-208054 27343213

[30] Armstrong B. Models for the relationship between ambient temperature and daily mortality. Epidemiology. 2006;17:624–31. 10.1097/01.ede.0000239732.50999.8f 17028505

[31] Gasparrini A. Distributed Lag Linear and Non-Linear Models in R: The Package dlnm. J Stat Softw. 2011;43(8):1–20. 22003319 PMC3191524

[32] Simon N.W. Generalized Additive Models: An Introduction with R. Chapman & Hall; 2006.

[33] Altman DG, Bland JM. Education and debate: Statistics Notes: Interaction revisited: the difference between two estimates. BMJ. 2003;326(January):219. 12543843 10.1136/bmj.326.7382.219 PMC1125071

[34] Lavigne E, Gasparrini A, Wang X, Chen H, Yagouti A, Fleury MD. Extreme ambient temperatures and cardiorespiratory emergency room visits: assessing risk by comorbid health conditions in a time series study. Environmental health. 2014;13:5 10.1186/1476-069X-13-5 24484632 PMC3922624

[35] Petrofsky JS. The Effect of Type-2-Diabetes-Related Vascular Endothelial Dysfunction on Skin Physiology and Activities of Daily Living. J Diabetes Sci Technol. 2011;5(3):657–67. 10.1177/193229681100500319 21722580 PMC3192631

[36] Kenny GP, Sigal RJ, Mcginn R. Body temperature regulation in diabetes. Temperature. 2016;3(1):119–45. 10.1080/23328940.2015.1131506 PMC4861190 27227101

[37] Modesti PA, Morabito M, Bertolozzi I, Massetti L, Panci G, Lumachi C, et al Weather-Related Changes in 24-Hour Blood Pressure Profile Effects of Age and Implications for Hypertension Management. Hypertension. 2006;47:155–61. 10.1161/01.HYP.0000199192.17126.d4 16380524

[38] Keatinge WR, Coleshaw SRK, Cotter F, Mattock M, Murphy M, Chelliah R. CLINICAL Increases in platelet and red cell counts, blood viscosity, and arterial pressure during mild surface cooling: factors in mortality from coronary and cerebral thrombosis in winter. Br Med J. 1984;289(November):1405–8. 6437575 10.1136/bmj.289.6456.1405 PMC1443679

[39] Sun Z. Cardiovascular responses to cold exposure. Front Biosci. 2011;2(27):495–503. 10.2741/e108 PMC2826836 20036896

[40] Hong YC, Kim H, Oh SY, Lim YH, Kim SY, Yoon HJ, et al Association of cold ambient temperature and cardiovascular markers. Sci Total Environ. 2012;435–436:74–9. 10.1016/j.scitotenv.2012.02.070 22846766

[41] Keatinge WR, Coleshaw SRK, Easton JC, Cotter F, Mattock MB, Chelliah R. Increased platelet and red cell counts, blood viscosity, and plasma cholesterol levels during heat stress, and mortality from coronary and cerebral thrombosis. Am J Med. 1986;81(5):795–800. 3776986 10.1016/0002-9343(86)90348-7

[42] Basu R. High ambient temperature and mortality: A review of epidemiologic studies from 2001 to 2008. Environ Heal A Glob Access Sci Source. 2009;8(1). 10.1186/1476-069X-8-40 PMC2759912 19758453

[43] Guo Y, Gasparrini A, Armstrong BG, Tawatsupa B, Tobias A, Lavigne E, et al Temperature variability and mortality: A multi-country study. Environ Health Perspect. 2016;124(10):1554–9. 10.1289/EHP149 27258598 PMC5047764

[44] Lim Y-H, Hong Y-C, Kim H. Effects of diurnal temperature range on cardiovascular and respiratory hospital admissions in Korea. Sci Total Environ. 2012 2 15;417–418:55–60. 10.1016/j.scitotenv.2011.12.048 22281041

[45] Liang W-M, Liu W-P, Kuo H-W. Diurnal temperature range and emergency room admissions for chronic obstructive pulmonary disease in Taiwan. Int J Biometeorol. 2009 1;53(1):17–23. 10.1007/s00484-008-0187-y 18989710

[46] Xu Z, Huang C, Su H, Turner LR, Qiao Z, Tong S. Diurnal temperature range and childhood asthma: a time-series study. Environmental Health 2013;12;12 10.1186/1476-069X-12-12 23374669 PMC3599100

[47] Song G, Chen G, Jiang L, Zhang Y, Zhao N, Chen B, et al Diurnal temperature range as a novel risk factor for COPD death. Respirology. 2008 11;13(7):1066–9. 10.1111/j.1440-1843.2008.01401.x 18922144

