# Supplementary material for: Short-term association between ambient temperature and acute myocardial infarction hospitalizations for diabetes mellitus patients: A time series study
Source: PLoS Med. 2018 Jul 17;15(7):e1002612. doi: 10.1371/journal.pmed.1002612 (PMC6049878; doi:10.1371/journal.pmed.1002612)
Supplement: S1 Text — (PDF) [file pmed.1002612.s001.pdf]

PROPOSED RESEARCH PROJECT

a) Title:

Meteorological risk factors for hospital admissions among patients with Diabetes Mellitus in Hong Kong.

b) Introduction:

The prevalence of diabetes mellitus (DM) has been increasing worldwide, and it is estimated that 366 million people have the disease [1]. The increase in prevalence has been particularly strong in East Asia where diabetes tends to occur at younger ages and at a much lower mean BMI when compared to western countries [1]. In Hong Kong an estimated 9.38% of the population had DM in 2001, with 46.7% of these having not been diagnosed [1]. The prevalence is slightly less that Japan, Canada and the United States, and slightly more than China, Korea, Australia and the U.K.[1]. The mean diabetes-related expenditure per DM patient in Hong Kong was US$2,059 in 2011 [1]. An international study found that, in addition to vascular diseases, diabetes was associated with increased mortality from several types of cancer, renal disease, pneumonia, liver disease, chronic obstructive pulmonary disease (COPD), falls and suicide, and that 50 year olds diagnosed with diabetes had a remaining life expectancy about 6 years less than for non-diabetics [2].

An association between ambient temperature and mortality/morbidity has been established by previous studies in regions around the world with higher rates of both being observed during periods of extreme heat or extreme cold [3-8]. However the strength of these associations and the threshold temperatures above (below) which heat (cold) effects become apparent varies geographically. Studies have shown that circulation to the skin [9] and sweating response [10] is impaired in diabetics. Hospital admissions among diabetics have been found to be more sensitive to heat wave effects [11]. A study on the relationship between inflammatory and coagulation responses to cold temperatures in patients with coronary disease [12] found that the association of several blood parameters with temperature was stronger in CHD patients who had diabetes vs. those who didn't and the authors concluded that diabetics were a susceptible subgroup which reacted more strongly to cold temperatures [12]. Our previous study on hot weather effects on mortality [3] estimated that deaths due to diabetes were 9.3% higher on days with a mean temperature of 31.2C vs a day with a mean temperature of 28.2C during the hot season in Hong Kong. This was not statistically significant however due to the small number of deaths with diabetes listed as the principal cause of death. Our study on cold weather and mortality [4] found a cumulative relative risk for diabetes deaths = 1.84 (95% CI = 1.42, 2.38, $p < .001$) for a 10C lower temperature over a 20 day lag period during the cool season in Hong Kong. This was the strongest association observed for all of the common causes of deaths in Hong Kong [4].

Our study looking at associations between weather and public hospital admissions in Hong Kong [13] found that hospitalizations due to respiratory and infectious diseases rose during very hot weather, while admissions for circulatory, respiratory and infectious disease rose during cold weather. This analysis did not examine the influence of co-morbidities on these associations. Recent increases in both DM prevalence and the frequency of extreme high temperatures pose serious public health threats particularly if those with DM are more sensitive to variations in meteorological conditions. A study specifically looking at the relationship between hospital admissions among those diagnosed with diabetes and meteorological variables is needed in order to determine whether the thermoregulation problems noted in diabetics leads to greater sensitivity of this group to extreme temperatures in a sub-tropical climate, and to aid in the patient management.

c) Aims and Hypotheses to be Tested:

- Our aim is to develop a statistical model relating daily hospitalizations among subjects with a

prior diagnosis of DM to recent meteorological and environmental conditions, including temperature, relative humidity, wind speed, solar radiation and pollutant levels.

- We hypothesize that hospitalizations among diabetics will be more sensitive to high temperatures and high humidity in the hot season than those among non-diabetics.

d) Plan of Investigation:

(i) Subjects

All admissions to Hong Kong Hospital Authority hospitals not due to external causes for the years from 2002-2011 from among subjects with a prior diagnosis of DM between 1998 and 2011 will be included in the models for diabetics. Admissions among those without a previous diagnosis of diabetes will also be included as a comparison group.

(ii) Methods

Distributed lag non-linear models with daily hospitalizations as the outcomes will be used to estimate the association between recent meteorological conditions and morbidity.

(iii) Study design

This will be a retrospective cohort time series (daily) study.

(iv) Data processing and analysis

Data

We currently have a dataset containing the following variables for all hospital admissions to Hong Kong public hospitals from 1998-2011: unique patient id, date of admission, date of discharge, death status, length of stay, gender, age, district of residence, hospital, and first 15 diagnosis codes (ICD-9), available for analysis. We also currently have meteorological data obtain from the Hong Kong Observatory, including mean daily temperature, relative humidity, solar radiation and wind speed for Hong Kong from 2002-2011, and daily pollutant data from the Hong Kong Environmental Protection Department (EPD) including nitrogen dioxide (NO2), sulphur dioxide (SO2), ozone (O3) and respirable suspended particulates (RSP). We will identify all patients with a diagnosis of diabetes (ICD-9 250.xx) in any of the first 125 diagnosis codes, and all admissions for these patients in the current or subsequent time periods will be considered as admissions among diabetics. For example consider a patient with 3 separate admissions, the 1st admission has no diagnosis of diabetes in any of the first 15 codes, the 2nd admission has a diagnosis of diabetes in 2nd diagnosis code, and the 3rd admission has no diabetes diagnosis. The 2nd and 3rd admissions, but not the 1st, for this patient would be considered as admissions among diabetics. Daily time series of the following outcomes with then be created among diabetics: all admissions not due to external causes (ICD-9 0-799), circulatory admissions (ICD-9 390-459), heart failure (HF; ICD-9 428.xx), acute myocardial infarction (AMI; ICD-9 410.xx), respiratory admission (ICD-9 460-519), COPD (ICD-9 490-496, excl. 493), asthma (ICD-9 493.xx), and pneumonia and influenza (ICD-9 480-487). These outcomes will also be created for admissions among non-diabetics for the purposes of comparison.

Statistical Analysis

Distributed lag non-linear models (DLNM) [Armstrong], an extension of Generalized Additive Models, will be used to assess the associations between meteorological variables and the daily hospitalization outcomes while taking into account non-linear and lagged effects, Daily mean temperature, relative humidity, solar radiation and wind speed will be modelled using splines with

5 degrees of freedom each for the non-linear effect of each variable and the lag structure. The maximum lag considered will be 28 days. Daily levels of pollutants, including NO2, RSP, SO2, and O3 will be modelled using splines with 5 degrees of freedom for non-linear effect, 3 degrees of freedom for the lag effects and maximum lags of 10 days. Long term trends will be controlled using a smooth term with maximum degrees of freedom of 10 (1 per year). Seasonality will be controlled using a smooth term for day of the year (1, 2, …365 or 366 for leap years) with maximum of 5 degrees of freedom. Day of the week and holiday effects will be controlled using indicator variables. The R statistical software package version 3.0.2 will be used for all analyses with the packages mgcv() [Wood] and dlnm () [Armstrong] being used for the modelling. The mgcv() package uses cross-validation to select the appropriate df for each non-linear term given a maximum df supplied by the user. The fit of the model will be examined by checking the partial autocorrelation of the residuals, normality of residuals, and scatterplots of residuals vs. predicted values. Adjusted cumulative relative risks and 95% confidence intervals for hospitalization risk corresponding to changes in meteorological and pollutant variables will be estimated from the models with the cumulative effect being estimated out to the maximum lag at which the observed effect is significant. For each outcome we will also perform subgroup analysis by age group (<65, 65-74, 75-84, and 85+) and gender.

Sample size

Among 6,484,457 hospitalizations with a primary diagnosis of non-external causes (ICD-9 0-799) in the HA data from 2002-2011, 1,347,891 (20.8%) had a concurrent or prior diagnosis of DM. Among the 5,997,414 of these admissions in subjects ≥ 15 years of age, 4,652,701 (22.4%) had a concurrent or prior diagnosis of DM. The corresponding DM proportions for specific principal diagnoses are: heart failure 60,538 (35.7%) of 169,441, AMI 16,566 (30.8%) of 53,786, HS 9,302 (16.0%) of 58,084, IS 41,542 (29.8%) of 139,318, respiratory 150,074 (15.3%) of 979,458, COPD 73,733 (11.9%) of 366,780, asthma 6,666 (13.7%) of 48,589, and P&I 67,128 (19.9%) of 336, 960. The large number of admissions for these diagnoses with DM and the fact that we are using a 10 year series of data should provide excellent precision for the resulting estimates of relative risks of hospitalization corresponding to changes in temperature and other meteorological variables.

[Word Court: 2128]

e) Existing Facilities:

The School of Public Health and Primary Care of the Chinese University of Hong Kong has the necessary computing facilities and software to carry out the analyses.

f) Justification of Requirements:

A research associate biostatistician will be needed to perform the complex data management, data cleaning, and the analyses for the project. The RA will also assist with the literature review and the process of writing the grant report and the papers for journal submission.

g) Purpose and Potential:

The results of this study will help to determine whether hospitalizations among people with diabetes are particularly sensitive to adverse environmental conditions compared to hospitalizations among

those who do not have a previous diagnosis of diabetes. This information will be useful for researchers studying the physiological effects of diabetes and those studying the relationship between meteorological conditions and health outcomes. This information will also help clinicians in providing patient care and advice to diabetics. The models developed for daily hospitalizations among diabetics can be used to identify combinations of meteorological and environmental conditions which are particularly threatening to DM patients.

h) Key References:

1. Ma RC, Chan, JC, Type 2 diabetes in East Asians: similarities and differences with populations in Europe and the United States. Ann NY Acad Sci 2013; 1281(1): 64-91.
2. Seshasai SR, Kaptoge S, Thompson A, et al. Diabetes mellitus, fasting glucose, and risk of cause-specific death. N. Engl. J. Med. 2011;364:829–841.
3. Chan EYY, Goggins WB, Kim JJ, Griffiths, A study of intracity variation of temperature-related mortality and socioeconomic status among the Chinese population in Hong Kong. *Journal of Epidemiology and Community Health* 2010. Epub. DOI: 10.1136/jech.2008.085167
4. Goggins WB, EY Chan, C Yang, M Chong, Associations between mortality and meteorological and pollutant variables during the cool season in two Asian cities with sub-tropical climates: Hong Kong and Taipei. Environmental Health 2013; 12:59 doi: 10.1186/1476-069X-12-59.
5. Curriero FC, Heiner KS, Samet JM, Zeger SL, Strug L, Patz JA. 2002. Temperature and mortality in 11 cities of the eastern United States. Am J Epidemiol 155: 80–87.
6. McMichael AJ, Wilkinson P, Kovat SJ. 2008. International study of temperature, heat and urban mortality: the 'ISOTHURM' project. Int J Epidemiol 37: 1121–1131.
7. Kan HD, Jia J, Chen BH. Temperature and daily mortality in Shanghai: a time-series study. Biomed Environ Sci 2003;16:133e9.
8. Chung JY, Honda H, Hong YC, Pan XC, Guo YL, Kim H, Ambient temperature and mortality: An international study in four capital cities in East Asia. *Sci Tot Environ* 2009; 408: 390-396.
9. Petrofsky JS, The effect of type-2-diabetes-related vascular endothelial dysfunction on skin physiology and activities of daily living. J Diabetes Sci Technol 2011; 5(3): 657-667.
10. Kenney GP, Yardley J, Brown C, Sigal RJ, Jay O, Heat stress in older individuals and patients with common chronic diseases, CMAJ 2010; 182 (10): 1053-1060.
11. Semenza JC, McCullough JE, Flanders WD, et al. Excess hospital admissions during the July 1995 heat wave in Chicago. *Am J Prev Med* 1999;16:269-77.
12. Hampel R, Breitner S, Ruckerl R, Frampton MW, Koenig W, Phipps RP, Wichmann HE, Peters A, Schneider A, Air temperature and inflammatory and coagulation response in men with coronary or pulmonary disease during the winter season. *Occup Environ Med 2010;67:408-416*
13. Chan EY, Goggins WB, Yue JS, Lee P, Hospital admissions as a function of temperature, other weather phenomena and pollution levels in an urban setting in China. *Bulletin of the World Health Organization* 2013; 91(8): 576-584.
14. Wood, S. (2006) Generalized Additive Models An Introduction with R. Chapman and Hall/CRC; Taylor and Francis Group
15. Armstrong B (2006) Models for the relationship between ambient temperature and daily mortality. Epidemiology 17: 624-31.
