# Supplementary material for: Short-term association between ambient temperature and acute myocardial infarction hospitalizations for diabetes mellitus patients: A time series study
Source: PLoS Med. 2018 Jul 17;15(7):e1002612. doi: 10.1371/journal.pmed.1002612 (PMC6049878; doi:10.1371/journal.pmed.1002612)
Supplement: S2 Text — (DOCX) [file pmed.1002612.s002.docx]

**Technical details of the regression models**

Exposure variables, including daily mean temperature, mean relative humidity (RH), wind speed, solar radiation and daily mean level of the four air-pollutants were included in the initial model with the daily number of AMI admissions for the DM and non-DM group as the outcome variables. Wind speed was square root-transformed while PM10 was log-transformed to reduce skewness. Meteorological and air-pollutants were all modelled using crossbasis() functions in dlnm() package of R (33). The cross-basis is a two dimensional functional space created by combining two basis functions, one for specifying the relationship between the outcome and the predictor, for example mean daily temperature, and the other representing the delayed effects, in our case specified as lag days (33). Thus these cross-basis functions can represent the three-dimensional relationship between predictor variable, lag, and the outcome, in our case daily counts of AMI hospitalizations. We used natural cubic splines, “ns” in the dlnm() package, to model both the potentially non-linear relationship between the predictor and outcome and the distributed lag curve. These cross-basis terms were then incorporated into Poisson Generalized Additive Models (GAMs) which also included natural cubic spline terms (34) for modeling variables for which non-linearity was possible but for which lagged effects were not a concern, including same-day rainfall, seasonality (included as day of the year: 1, …, 365 (366)), and long term time trend (included as day of study: 1, …,3227). The GAMs also included indicator variables for categorical variables including day of the week, and public holidays. The initial model described is shown as below:

Log(E[daily no. of AMI admissions in DM(non-DM group)]) =

cb(temp, df=3; lag, df=3) + cb(humid, df=3; lag, df=3) + cb(sqrt.wind_speed, df=3; lag, df=3) + cb(solrad, df=3; lag, df=3) + cb(air pollutants, df=3; lag, df=3) + s(sqrt.Rain, maximum df=2) + s(long term trend, maximum df=10) + s(seasonal trend, maximum df=4) + factor(DOW) + factor(Holiday)

*cb: crossbasis of independent variables built using the dlnm() package in R*

*s(): natural cubic spline of the variable from the mgcv() package for GAM in R*

*factor(): creates indicator variables of categorical independent variables*

*air pollutants: PM_10_, SO_2_, NO_2_ or O_3_*

Long term trend: Day of study (1,2,3…,3227)

Seasonal trend: Day of year (1,2,3, …,365/366)

DOW: Day of week (1,2,3,…,7)

Maximum lags of 30 days for meteorological factors, and 10 days for air-pollutants and a degree of freedom (df) of 3 for both lag and exposure were adopted for the models. The dfs were chosen by comparing the results of the initial models with different dfs, starting from df = 6 to 3. The RR-lag plot shows how relative risk comparing two levels of exposures changes along with time, which means the lag in time series study. Based on the standard setting of 1 df for each year for long term trend and 7 df for each year (i.e. df=4 for each season with 6 months), the maximum df allowed for rainfall, long term trend and seasonal trend were 2, 10 and 4, respectively. For the natural cubic spline terms, s(), the mgcv package chooses the actual df used through minimizing the generalized cross-validation (GCV) score which effectively chooses the df to minimize prediction error while avoiding overfitting the sample of data (34).

As an illustration of how the cross-basis terms represent the three-dimensional association between outcome, predictor, and lag, figure S2.1 shows a three-dimensional contour plots from our model for the AMI and temperature association during the cold seasons for the DM patients.

Figure S2.1: Three dimensional plot of relative risk of AMI admissions vs. mean daily temperature (reference temperature = 24℃) and lag days for DM patients, November to May, 2002-2011.


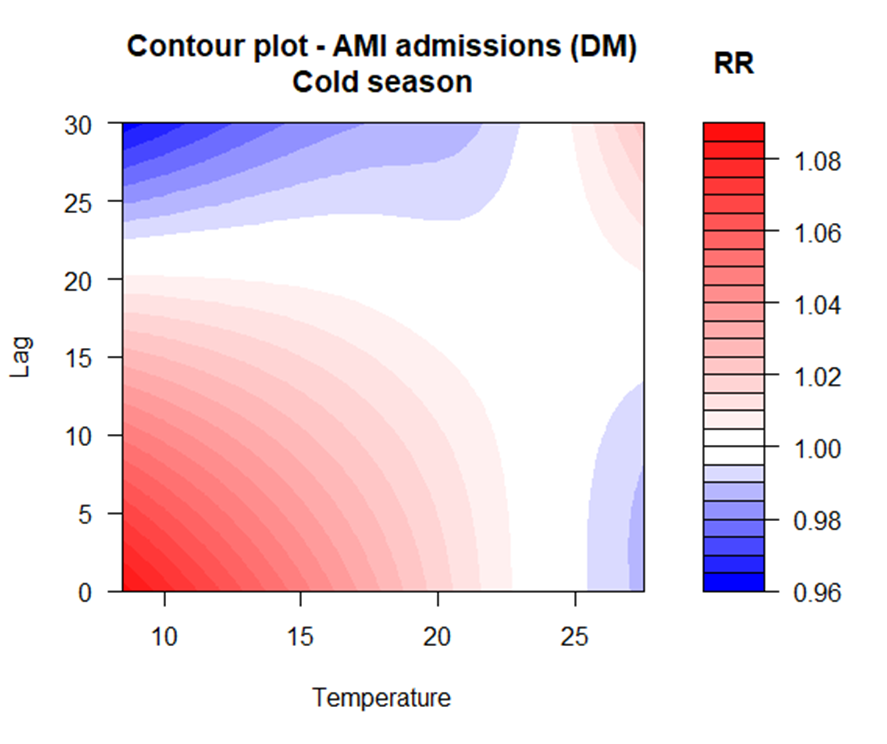


From the plot we can see that the relative risks for at low temperatures are highest at lag 0 (same day), but continue to be elevated for an addition three weeks with the associations gradually diminishing in strength.

During the model selection process, environmental variables that did not show obvious associations with AMI admissions were dropped from the model one at a time. Associations were examined using a Relative Risk (RR)-exposure plot. If the RR changed considerably with the study exposure, such as showing a linear trend or a u-shaped association, the exposure variable was considered to be associated with AMI admissions and will be kept in the model, otherwise would be dropped. During the modelling procedure, whenever a variable was dropped from the model, the temperature-AMI association was compared to that of the model before the particular variable was dropped to double check for multicollinearity. Same day rainfall, long term time trend, seasonal trend, day-of-week and holiday were adjusted in the whole modeling process.

The final model was as below:

Log(E[daily no. of AMI admissions in DM( non-DM group)]) =

cb(temp, df=3; lag, df=3) + cb(humid, df=3; lag, df=3) + cb(sqrt.wind_speed, df=3; lag, df=3) + cb(NO_2_, df=3; lag, df=3) + s(sqrt.Rain, maximum df=2) + s(long term trend, maximum df=10) + s(seasonal trend, maximum df=4) + factor(DOW) + factor(Holiday)

The length of the delayed effects was estimated based on the visual observation in the RR-lag plots. The lag-value where the RR curve cuts RR = 1.0 (the x-axis) or where the RR curve becomes stable (parallel to the x-axis) was taken as the end point of the delayed effect. The RR-lag plot for the cold season temperature-AMI association for DM patients is shown in Figure S2.2.

Figure S2.2 Plot of relative risk for AMI hospitalizations for DM patients at 12℃ vs. 24℃, November-April, 2002-2011.


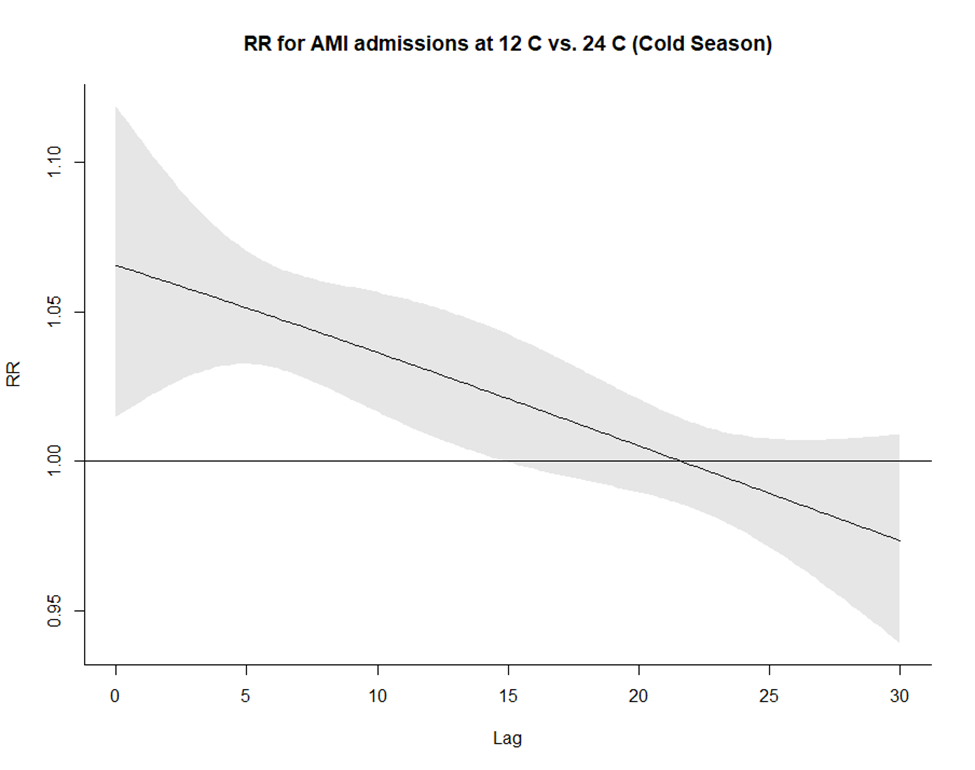


From the plot we can see that the association with cold temperature is strongest at lag 0 but persists for about three weeks. The cumulative RR-lag plot, which for each lag shows the cumulative RR from lag 0 up to the designated lag, is shown in figure S2.3.

Figure S2.3: Plot of cumulative relative risk for AMI hospitalizations for DM patients at 12℃ vs. 24℃, November-April, 2002-2011.

From his plot we can see that excess risk due to cold temperatures stops rising after a lag of about three weeks. Essentially the effect of a day with very low temperatures for DM appears to be an immediate rise in risk of AMI which diminishes over time but persists for about three weeks. To capture the overall association with temperatures the cumulative AMI-temperature associations were displayed for lags 0-22 in figure 1 in the main text.

In general, cumulative relative risk (RR) of admissions were estimated by comparing the number of admissions during extreme temperature to that during a reference temperature cumulatively over the number of lagged-day identified. The temperature at the 97^th^ percentile during hot season was taken to represent extreme high temperature while that at the 3^rd^ percentile during the cold season represented extreme low temperature. The reference points were chosen based on nature of association observed. The temperature associated with the lowest risk, the minimum morbidity temperature (MMT), for a U-shaped association or the median temperature in a linear association were used as the reference value for comparison. In a RR-temperature plot, RR curve that obviously deviated from RR = 1.0 (the x-axis) implied a change of admission risks associated with temperature. The farther the RR-curve deviated from RR=1.0, the larger the effect. The association was considered statistically significant if the 95% confidence interval of RR did not include RR =1.0.

**Calculation of the Relative Risk Ratio (RRR)**

To compare the RRs for AMI admissions between the DM and non-DM groups, we examined the exposure-response curves for each group and calculated the relative risk ratio (RRR) and the corresponding 95% confidence interval (CI) using the approach suggested by Altman and Bland in 2003 (35), using the estimated RRs and the corresponding 95% Confidence Interval (CI) for the two groups.

Below are the formulae for calculating the RRRs:

- Let RR for group 1 = RR1 (with 95%CI) , RR for group 2 = RR2 (with 95%CI)
- d = Log RR1 – Log RR2
- SE (d) = square root [ { Width(CI of Log RR1)/ (2x 1.96) }^2^ + { Width(CI of Log RR2)/ (2x 1.96) }^2^ ]
- RRR = exp ( d )
- 95% Confidence Interval of RRR = exp ( d + 1.96 x SE (d)) to exp ( d – 1.96 x SE (d))

An example showing the calculation of RRR comparing DM and non-DM among all AMI admission in the Cold season (Result in Table 3) [Note: Only 2 decimal places were considered in this example, i.e. the results here are slightly different from Table 3]

RR1 = 2.10 (1.62, 2.72), RR2 = 1.43 (1.21, 1.69)

d = Log (2.10) – Log (1.43) = 0.74 – 0.36 = 0.38

SE (d) = Square root [ {(Log 1.62 – Log 2.72) / (2x 1.96) } ^2^ + {(Log 1.21 – Log 1.69) / (2x 1.96) } ^2^ ] = square root [ 0.13^2^ + 0.09^2^ ] = 0.16

RRR = exp (d) = exp (0.38) = 1.47

95% CI of RRR = exp (1.47 - 1.96 x 0.16) to exp ( 1.47 + 1.96 x 0.16) = 1.07 to 2.00

**Table S2.1 Correlation table of environmental variables in final models in seasons.**

| Cold season (November to April) N = 1812 | | | | |
| --- | --- | --- | --- | --- |
|  | Mean Temperature | Mean relative humidity | Square-root mean wind speed | Mean NO2 |
| Mean Temperature | 1.00 | 0.32^**^ | -0.40^**^ | -0.04 |
| Mean relative humidity | - | 1.00 | -0.23^**^ | -0.30^**^ |
| Square-root mean wind speed | - | - | 1.00 | -0.35^**^ |
| Mean NO2 | - | - | - | 1.00 |
| Hot season (May to October) N = 1840 | | | | |
|  | Mean Temperature | Mean relative humidity | Square-root mean wind speed | Mean NO2 |
| Mean Temperature | 1.00 | -0.13^**^ | -0.26^**^ | -0.28^**^ |
| Mean relative humidity | - | 1.00 | 0.05^*^ | -0.33^**^ |
| Square-root mean wind speed | - | - | 1.00 | -0.25^**^ |
| Mean NO2 | - | - | - | 1.00 |
| **. Correlation is significant at the 0.01 level (2-tailed). | | | | |
| *. Correlation is significant at the 0.05 level (2-tailed). | | | | |
