# Supplementary material for: Short-term association between ambient temperature and acute myocardial infarction hospitalizations for diabetes mellitus patients: A time series study
Source: PLoS Med. 2018 Jul 17;15(7):e1002612. doi: 10.1371/journal.pmed.1002612 (PMC6049878; doi:10.1371/journal.pmed.1002612)
Supplement: S3 Text — (DOCX) [file pmed.1002612.s003.docx]

**Figure S3.1- S3.8 - Sensitivity analyses using Hot season data (May - Oct, 2002-2011)**


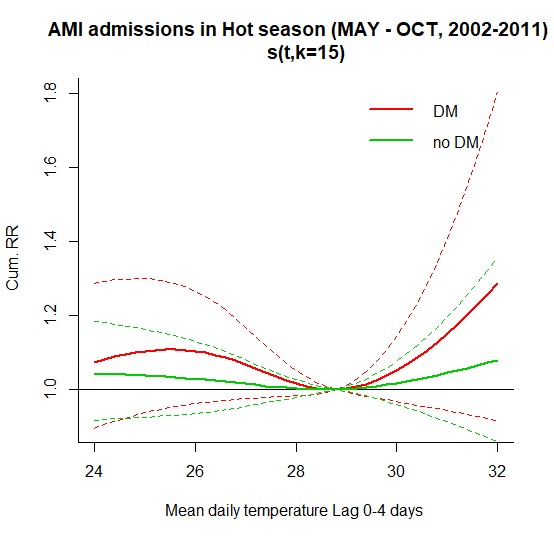


Figure S3.1 RR-temperature plot in hot season with degree of freedom (df = k-1 =4) for long term trend


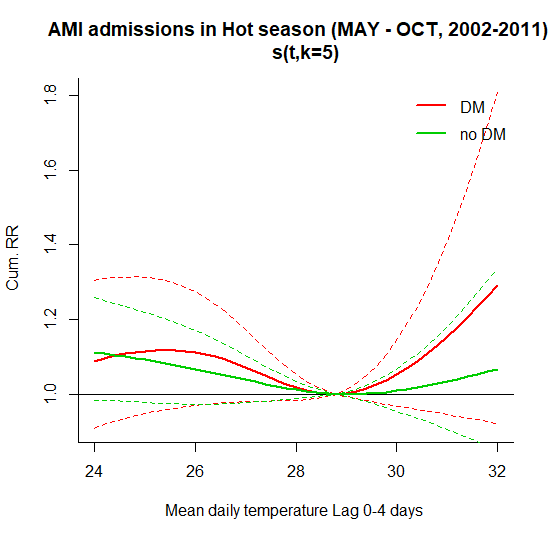


Figure S3.2 RR-temperature plot in hot season with degree of freedom (df = k-1 = 14) for long term trend


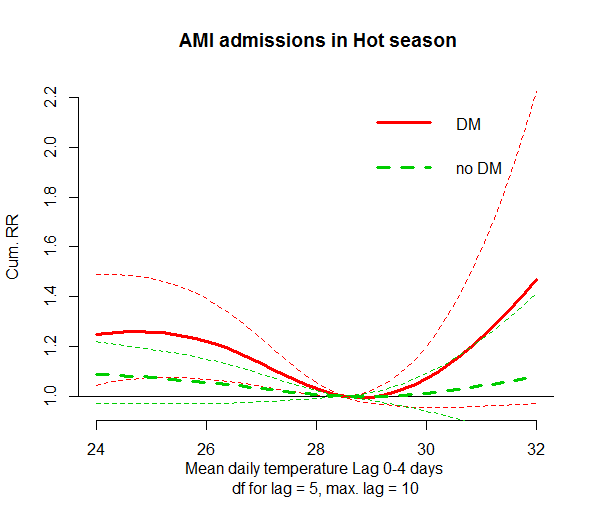


Figure S3.3 RR-temperature plot in hot season with degree of freedom for lag = 5


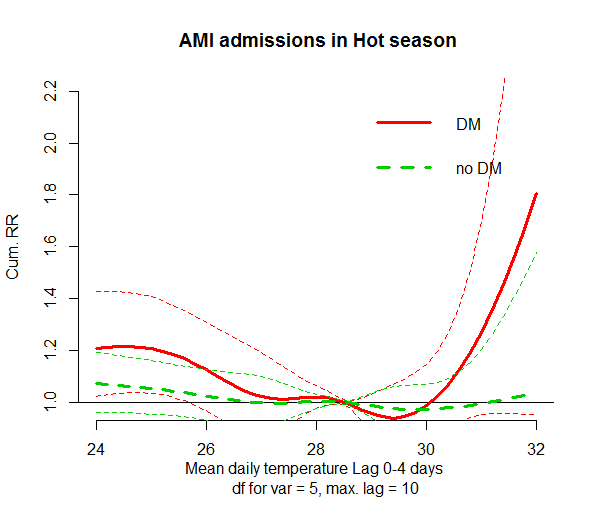


Figure S3.4 RR-temperature plot in hot season with degree of freedom for temperature = 5


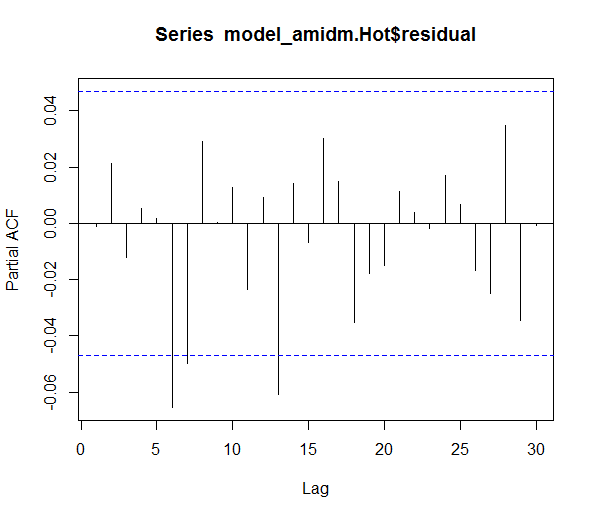


Figure S3.5 Partial Autocorrelation Function (PACF) plot in hot season for DM group.


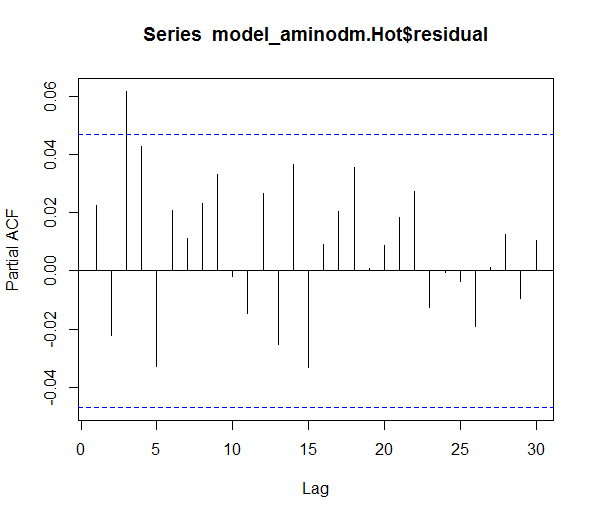


Figure S3.6 Partial Autocorrelation Function (PACF) plot in hot season for non-DM group.


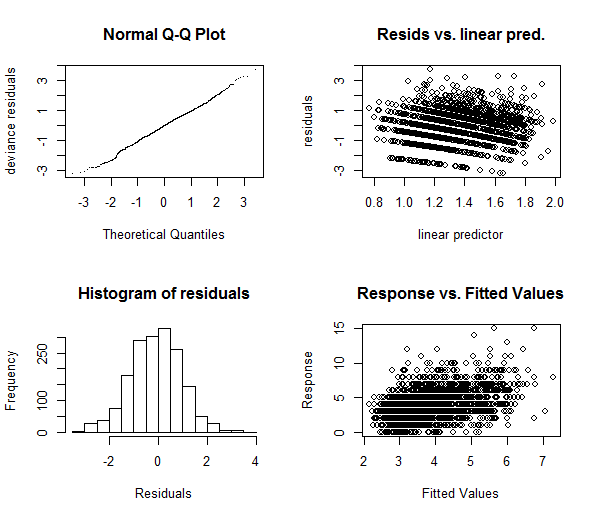


Figure S3.7 Residuals plots in hot season for DM group.


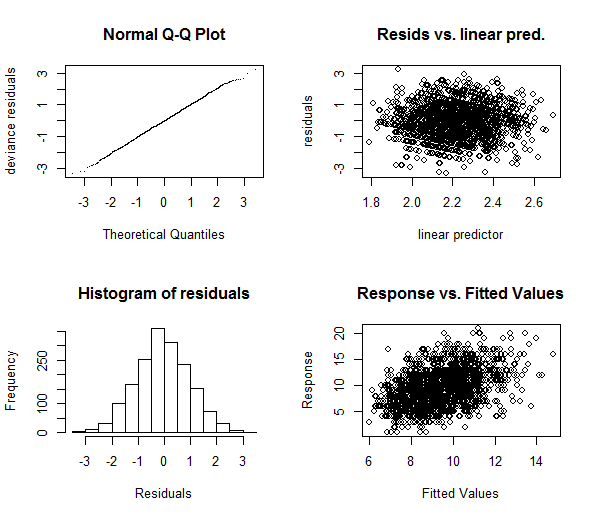


Figure S3.8 Residuals plots in hot season for non-DM group.

**Figure S3.9- S3.16 - Sensitivity analyses using Cold season data (Nov – Apr, 2002-2011)**


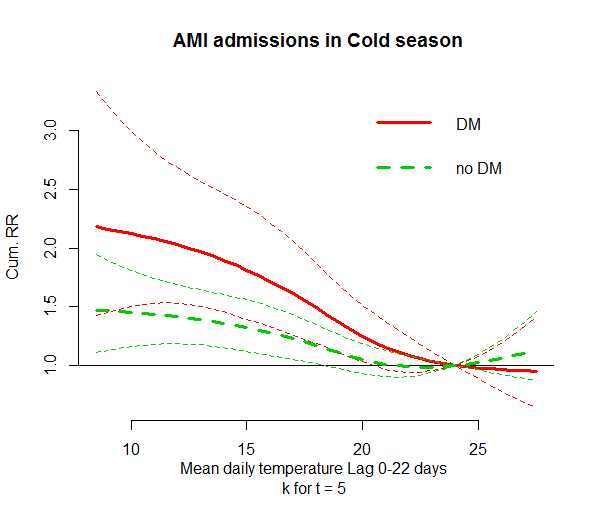


Figure S3.9 RR-temperature plot in cold season with degree of freedom (df = k-1 =4) for long term trend


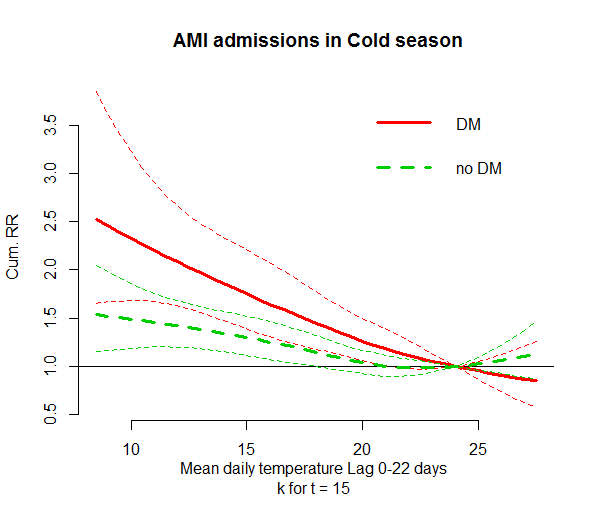


Figure S3.10 RR-temperature plot in cold season with degree of freedom (df = k-1 =14) for long term trend


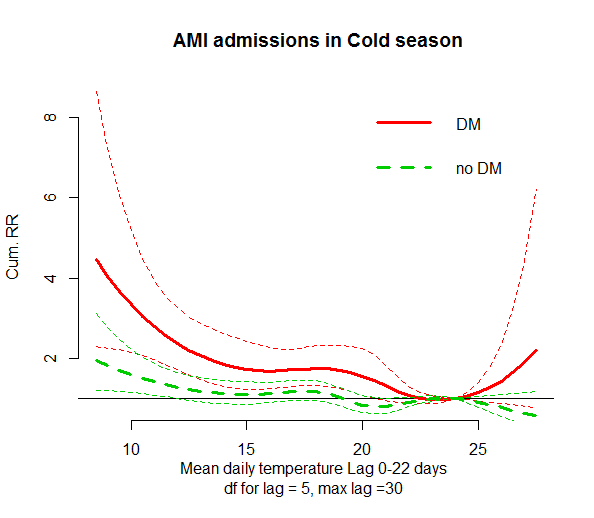


Figure S3.11 RR-temperature plot in cold season with degree of freedom for lag = 5


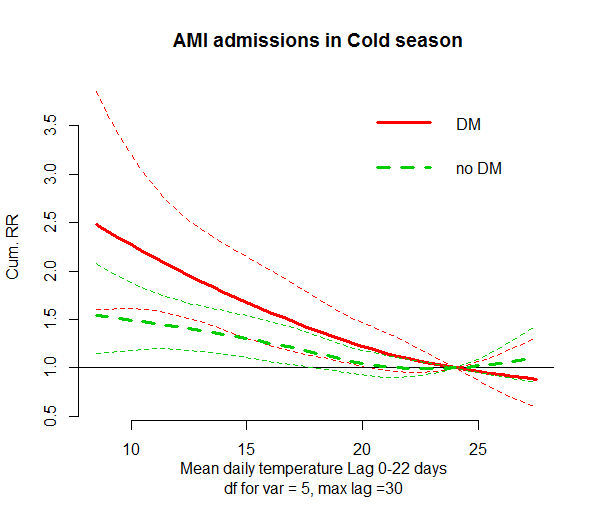


Figure S3.12 RR-temperature plot in cold season with degree of freedom for temperature = 5


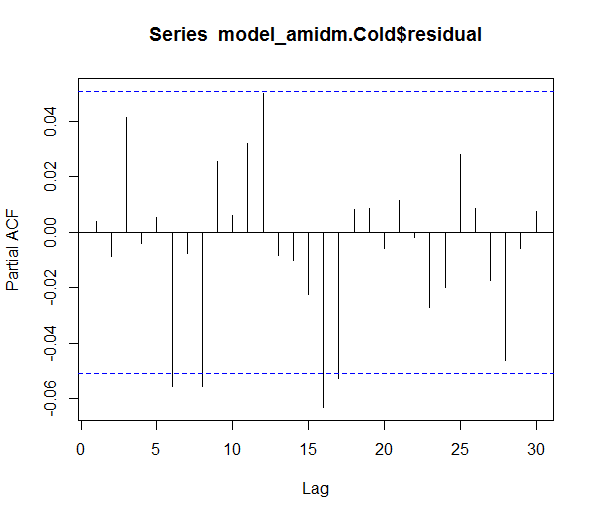


Figure S3.13 Partial Autocorrelation Function (PACF) plot in cold season for DM group.


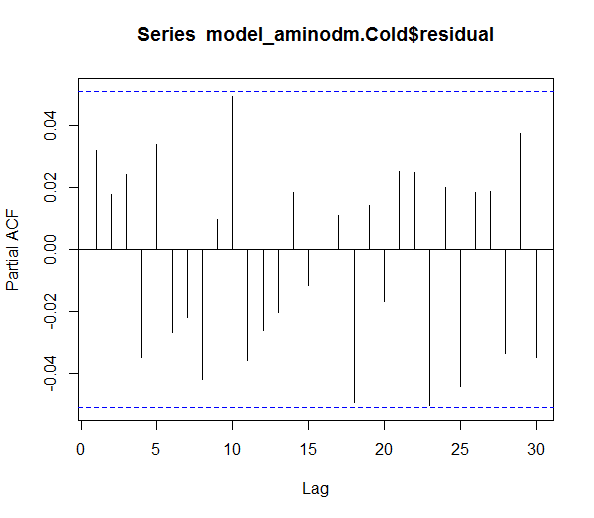


Figure S3.14 Partial Autocorrelation Function (PACF) plot in cold season for DM group.

|  |
| --- |
|  |
|  |


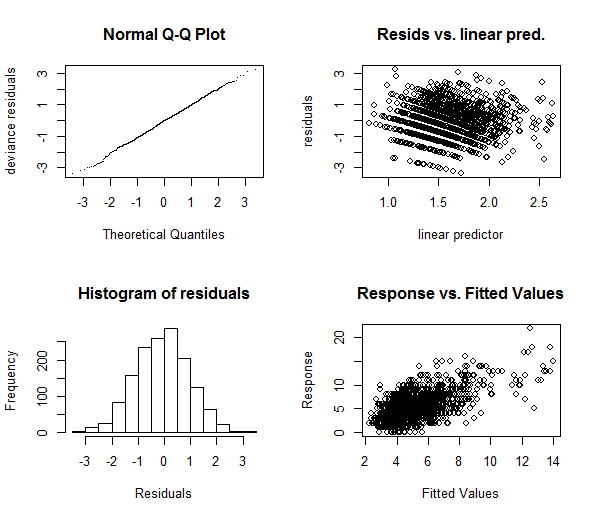


Figure S3.15 Residuals plots in cold season for DM group.


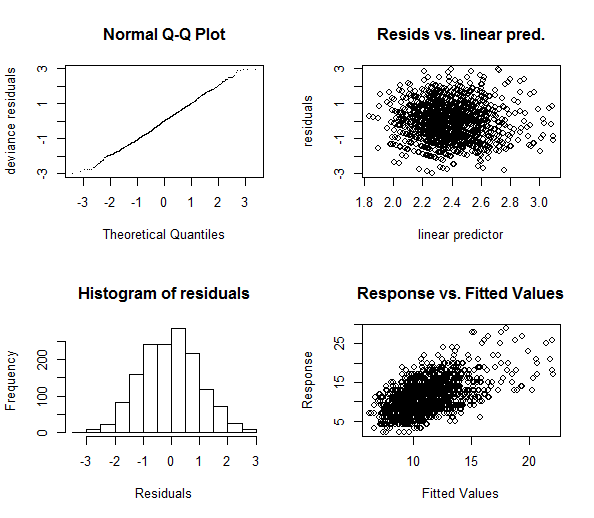


Figure S3.16 Residuals plots in cold season for non-DM group.
